# Supplementary material for: Systemic genome-epigenome analysis captures a lineage-specific super-enhancer for MYB in gastrointestinal adenocarcinoma
Source: Mol Syst Biol. 2025 Apr 15;21(6):696–719. doi: 10.1038/s44320-025-00098-1 (PMC12130324; doi:10.1038/s44320-025-00098-1)
Supplement: Supplementary file 1 — Appendix [file 44320_2025_98_MOESM1_ESM.pdf]

Appendix for

Systemic genome-epigenome analysis captures a lineage-specific super-enhancer for *MYB* in gastrointestinal adenocarcinoma

Fuyuan Li *et al*.

TABLE OF CONTENTS

Appendix Materials and Methods 2

References for Appendix Information 7

Appendix Table S1 9

Appendix Figures 11

Appendix Materials and Methods

Analysis of PCAWG and TCGA datasets

The list of gastrointestinal adenocarcinoma structural variants was downloaded from the Pan-Cancer Atlas of Whole Genomes (PCAWG) project (URL:https://dcc.icgc.org/releases/PCAWG/consensus_sv) (Consortium, 2020). Continuous 5 kb bins were tiled across the entire genome using BEDTools (Quinlan & Hall, 2010), and their duplication frequency in gastrointestinal adenocarcinoma were calculated. The adjacent bins were stitched together to form duplications and amplicons (duplication frequency ≥ 5). To focus on the focal duplication, we selected duplications that are less than 1 Mb in length for further analysis. TCGA ATAC-seq data was download from the NCI Genomic Data Commons data portal (URL: https://gdc.cancer.gov/about-data/publications/ATACseq-AWG) (Corces *et al*, 2018). We used the normalized bigWig data for Integrative Genomics Viewer presentation and we converted the DNA coordinates of the ATAC sites from hg38 to hg19 using the liftOver tool (Kent *et al*, 2002). For RNA expression analysis, we used GEPIA2 (Tang *et al*, 2019) for analyzing the RNA sequencing expression data of tumors and normal samples from the TCGA and the GTEx projects.

DepMap CRISPR screen analsyis

We analyzed the publicly available CRISPR screen results on cancer cell lines from the Broad Institute Dependency Map (DepMap) project (Tsherniak *et al*, 2017). The Spearman correlation between RNA expression results from the Cancer Cell Line Encyclopedia (CCLE) project and CERES, the CRISPR dependency score, was calculated as previously reported (Meyers *et al*, 2017). Also, we compared the correlation between the Broad CRISPR screen results and the Sanger Institute CRISPR screen results or the Broad shRNA screen results. Gene-level copy number data that is log_2 transformed with an absolute-count of 1; log_2(copy number/2). Inferred from WGS, WES or SNP array depending on the availability of the data type. All data were downloaded in September of 2023.

Motif enrichment analysis

ATAC-seq signal from COREAD and STAD was to narrow down DNA coordinates of enhancer e4 region. With the default parameters, the FIMO software (Grant *et al*, 2011) was used to identify transcription factor binding motifs from the JASPAR motif database (Castro-Mondragon *et al*, 2022) that are present in e4. Also, accessible chromatin region of e4 was subject to motif analysis using Transcription Factor Affinity Prediction (TRAP) web tools (Thomas-Chollier *et al*, 2011). The region was analyzed using JASPAR vertebrates as the matrix file, all human promoters as the background model, and Benjamini-Hochberg as the multiple test correction. We generated ChIP-seq data for MYB in HT-55 and SNU-719 cells in the present work. HOMER (Heinz *et al*, 2010) was used to analysis the motifs, HOMER script “findMotifsGenome.pl” was then performed for motif search with parameters –no motif and hg19. Besides, deeptools (Ramirez *et al*,

2016) (v3.5.1) and R package ChIPseeke (Wang *et al*, 2022; Yu *et al*, 2015) (v1.32.1) were used to annotate the ChIP-seq results.

Identification of predicted super-enhancers

H3K27ac ChIP-seq datasets were downloaded from the Gene Expression Omnibus (GEO) dataset. H3K27ac ChIP-seq fastq data were aligned to the hg19 genome assembly using Bowtie2 (Langmead & Salzberg, 2012) (v2.2.5). Peaks were called using MACS2(Zhang *et al*, 2008) (v2.2.7.1). BedGraph files were generated by MACS2, sorted, and normalized by the mean genome-wide bedGraph signal in each dataset. Bigwig tracks were generated from bedGraph using UCSC tool bedGraphToBigWig, and visualized by gTrack (Hadi *et al*, 2020) (v0.1.0) (https://github.com/mskilab-org/gTrack). Predicted enhancers were identified as enriched H3K27ac regions at least 2.5kb away from annotated transcription starting site (TSS) regions. We allowed enhancers within 15,000 bp to be stitched together. Predicted enhancers were then further divided into super-enhancers and typical enhancers using the ROSE (Whyte *et al*, 2013) algorithm.

Analysis of HiChIP and Hi-C data

HiChIP and Hi-C data was downloaded from the Gene Expression Omnibus (GEO) database. The sequencing reads were aligned to the hg19 human genome using the HiC-Pro pipeline (Servant *et al*, 2015), we the used the AllValidPairs output from HiC-Pro that lists all valid paired-end tags (PETs). Then, we used the hichipper pipeline (Lareau & Aryee, 2018) to call significant chromatin loops and used TCGA ATAC sites identified in COREAD and STAD cancer as pre-determined peaks for loop calling by hichipper. The loops supported by at least two PETs and associated with an FDR value < 0.05 were considered as significant loops. We next mapped the PETs to continuous 5kb bins within the regions of interest and presented the interactions as contact map using the gTrack (Hadi *et al.*, 2020) R package.

Super-enhancers nominated genes analysis

The expression level of Super-enhancers nominated 205 protein-coding genes analysis by GEPIA2 (Tang *et al.*, 2019) tools, including TCGA and GTEx project data. Gene Ontology (GO), KEGG and Reactome analysis were performed using the online DAVID tool (Sherman *et al*, 2022). Using H3K27ac HiChIP data in HT-55 and SNU-719 cell lines for identified the interactions between the super-enhancers and nominated protein-coding genes. Identification of oncogenes based on annotated information from COSMIC (https://cancer.sanger.ac.uk/census) (Sondka *et al*, 2018), OncoKB (https://www.oncokb.org/cancerGenes (Chakravarty *et al*, 2017) and ONGene (https://ongene.bioinfo-minzhao.org/) (Liu *et al*, 2017) databases.

RNA-seq

Total cells RNA was extracted using the Zymo Quick-RNA miniprep kit (Zymo, #R1055) according to the manual. PolyA mRNA was purified using NEBNext PolyA mRNA Isolation Module (NEB, #E3370S),

as per the manufacturer's instructions. RNA-seq libraries were prepared using the NEBNext Ultra Directional II RNA library prep kit and sequenced by Illumina/BGI with a 150 bp paired-end run configuration. Three biological replicates per sample.

RNA-seq analysis

Quality control was performed using the FastQC (v0.11.7) tool and the results were analyzed. Cleaned RNA-seq reads were mapped using STAR (Dobin *et al*, 2013) (v. 2.7.10) against the human genome (hg19). Uniquely mapped reads were quantified with HTSeq (Anders *et al*, 2015) (v.0.11.3) and protein-coding genes with nonzero read count (n = 20138) were included for downstream analysis. Differential expression of protein-coding genes was analyzed with these counts using Bioconductor package DESeq2 (Love *et al*, 2014) (v.1.40.1). Significantly differential expression was considered by setting adj$P < 0.05$ and FC $\geq$1.5 between sh*MYB* and shNC. Gene Ontology (GO) and KEGG analysis were performed using the online DAVID tool (Sherman *et al.*, 2022). The gene set enrichment analysis was performed according to the instructions (https://www.gsea-msigdb.org/gsea/index.jsp).

ChIP-seq and ChIP-qPCR

Chromatin-immunoprecipitation followed by massive parallel sequencing (ChIP-seq) was performed as previously described (Liu *et al*, 2021). In brief, 6 million cells were crosslinked with 1% formaldehyde and lysed. The chromatin extract was sonicated by the QSonica Q800R3 sonicator (25 minutes, 70% amplitude) and immunoprecipitated with anti-MYB antibody (Abcam, #ab45150), anti-HNF4A antibody (Abcam, #181604), and anti-EBF1 antibody (Millipore, AB10523), which were mixed with Dynabeads A and G (Invitrogen, #10002D/10004D). The immunoprecipitated DNAs were then used to construct the ChIP-seq library, following the protocol provided by the manufacturer and sequenced on Illumina Xten with the PE 150 method, and sequenced by Illumina NovaSeq. For chromatin immunoprecipitation (ChIP)-qPCR assays, we designed primers targeting MYB, EBF1 and HNF4A binding sites in e4. We also used sonicated genomic DNA to normalize variance in primer efficiency. All the primers are listed in **Table EV5**.

ChIP-seq analysis

Sequencing reads aligned to the hg19 human genome reference by Bowtie2 (v 2.2.5), and SAMtools(Li *et al*, 2009) (v1.6) was used to sort and index the aligned reads. PCR duplicates were marked and removed using Picard (v2.18.29) (https://github.com/broadinstitute/picard) MarkDuplicates function. MYB binding sites were called by MACS2 (Zhang *et al.*, 2008) (v2.2.6) using the default parameters (q value < 0.05). Bedgraph files generated with MACS2 were later converted to bigwig using UCSC bedGraphToBigWig tool, which was then visualized by Integrative Genomics Viewer (IGV) (Robinson *et al*, 2023). The H3K27ac HiChIP data of HT-55 and primary colonic epithelial cells were analyzed by chip-seq pipeline to obtain ChIP-seq-like H3K27ac signal. Gene Ontology (GO) and KEGG analysis of MYB ChIP-seq were

performed using the online DAVID (Sherman *et al.*, 2022) tool.

Genomic STARR-seq analysis

GP5d STARR-seq data was downloaded from GEO dataset, the accession number is GSM5454434. The analysis was carried out according to the previously reported methods(Sahu *et al*, 2022). In brief, the sequencing reads were aligned to the human genome (hg19) using Bowtie2 (Langmead & Salzberg, 2012) (v2.2.5, option –maxins 1000). Mapped read pairs were deduplicated using Picard (version 2.9.0) (https://github.com/broadinstitute/picard) and paired-end reads that had mapping quality < 20 or mapped in a discordant orientation were discarded using SAMtools (Li *et al.*, 2009) (v1.6). Then, the active enhancers were identified by calling the peaks from the STARR-seq–enriched RNA fragments against the plasmid input sample using MACS2 (Zhang *et al.*, 2008) (v2.2.6, -f BAMPE).

Site-directed deletion of motif sequence

The Q5® Site-Directed Mutagenesis Kit (NEB, #E0554) was used to generate deletions of the predicted motif sequences in the e4 region. Primers designed by using the NEB online design software, NEBaseChanger™, and the sequences are listed in **Table EV5**.

Plasmid transfection and lentiviral production

sgRNAs for CRSISPRi and CRSISPR-Cas9-mediated DNA motif cutting were cloned into the BsmBI (Thermo Fisher Scientific, #FD0454) sites of the lentiGuide-Pour (Addgene, #52963) lentiviral vector. sgRNAs for CRSISPRa were cloned into the BsmBI sites of the pXPR_502 (Addgene, #96923) lentiviral vector. The double-stranded oligonucleotide shRNA was cloned into the AgeI/EcoRI (NEB, #R3552L/R3101L) sites of the pLKO.1 (Addgene, #8453) lentiviral vector. All sgRNA and shRNA target sequences were listed in **Table EV5**. HEK293-FT cells were co-transfected with lentiviral packaging vectors in 10% FBS, 0.1% P/S DMEM high glucose medium. Plasmids transfection was performed according to the manual of CalPhos Mammalian Transfection Kit (Clontech, #631312). 24 h after transfection, the medium was replaced with fresh medium. 48h after transfection, the supernatant containing virus was collected and filtered through a 0.45 μm filter (Merck, #SLHP033R) to remove cells and debris. Virus then was added to cells or stored at -80 °C.

BI-6015 treatment

HNF4A antagonist BI-6015 (MedChemEpress, #HY-108469) was dissolved with DMSO into 5 mM storage solution, and stored at -80 °C. When needed, the storage solution was diluted into the corresponding concentration with working solution. The cells were treated for 48 h before tested with various assays.

BMS-986115 treatment

BMS-986115 (Notch inhibitor 1) (MedChemEpress, #HY-12860) was dissolved with DMSO into 5 μM storage solution, and stored at -80 °C. When needed, the storage solution was diluted into the corresponding

concentration with working solution. The cells were treated for 5-day before tested with various assays.

Quantitative RT-PCR

Total RNA was isolated using TRIzol (TAKARA, #9109). 1μg RNA was reverse transcribed into cDNA using LunaScript® RT SuperMix Kit (NEB, #E3010L). qPCR was performed with Luna® Universal qPCR Master Mix (NEB, #M3003E). The fold changes were normalized to *HPRT1* or *R28S*. The primers used for qPCR were listed in **Table EV5**.

Western Blot

Cells were harvested and washed in cold PBS for 3 times. Cells were lysed with NP40 lysis buffer (1% NP40, 150mM NaCl, 50mM Tris-HCl, pH 8.0) supplemented with protease inhibitors and sonicated with QSonica Q800R (pulse: 30s on/ 30s off, sonication time: 3 mins, amplitude: 50%). c-MYB (ProteinTech, #17800-1-AP, 1:1000), HNFA4 (CST, #3113S), anti-EBF1 antibody (Millipore, AB10523), and ACTB (CWBio, #CW0096M, 1:2000) antibodies were used.

Immunohistochemistry and EdU analysis

4% polyformaldehyde-fixed (Beyotime, #P0099), paraffin-embedded tumor sections (4-μm-thick) were dewaxed and rehydrated through graded alcohol to water before antigen retrieval, followed by treatment with 3% hydrogen peroxide. The sections were incubated with special antibody against Ki-67 (CST, #9449S) at 4°C diluted at 1: 400, subsequently stained with the DAB (Thermo Fisher Scientific, #34002) on the following day. Then, the sections were counterstained with hematoxylin (Beyotime, #C0107). For EdU staining, 10000 cells were seed into 96-well plate and cultured overnight. Then Staining analysis was performed using Cell-Light EdU Apollo488 In Vitro Kit (RIBOBIO, #C10310-3).

References for Appendix Information

Anders S, Pyl PT, Huber W (2015) HTSeq--a Python framework to work with high-throughput sequencing data. *Bioinformatics* 31: 166-169

Castro-Mondragon JA, Riudavets-Puig R, Rauluseviciute I, Lemma RB, Turchi L, Blanc-Mathieu R, Lucas J, Boddie P, Khan A, Manosalva Perez N *et al* (2022) JASPAR 2022: the 9th release of the open-access database of transcription factor binding profiles. *Nucleic Acids Res* 50: D165-D173

Chakravarty D, Gao J, Phillips SM, Kundra R, Zhang H, Wang J, Rudolph JE, Yaeger R, Soumerai T, Nissan MH *et al* (2017) OncoKB: A Precision Oncology Knowledge Base. *JCO Precis Oncol* 2017

Consortium ITP-CAoWG (2020) Pan-cancer analysis of whole genomes. *Nature* 578: 82-93

Corces MR, Granja JM, Shams S, Louie BH, Seoane JA, Zhou W, Silva TC, Groeneveld C, Wong CK, Cho SW *et al* (2018) The chromatin accessibility landscape of primary human cancers. *Science* 362

Dobin A, Davis CA, Schlesinger F, Drenkow J, Zaleski C, Jha S, Batut P, Chaisson M, Gingeras TR (2013) STAR: ultrafast universal RNA-seq aligner. *Bioinformatics* 29: 15-21

Grant CE, Bailey TL, Noble WS (2011) FIMO: scanning for occurrences of a given motif. *Bioinformatics* 27: 1017-1018

Hadi K, Yao X, Behr JM, Deshpande A, Xanthopoulakis C, Tian H, Kudman S, Rosiene J, Darmofal M, DeRose J *et al* (2020) Distinct Classes of Complex Structural Variation Uncovered across Thousands of Cancer Genome Graphs. *Cell* 183: 197-210 e132

Heinz S, Benner C, Spann N, Bertolino E, Lin YC, Laslo P, Cheng JX, Murre C, Singh H, Glass CK (2010) Simple combinations of lineage-determining transcription factors prime cis-regulatory elements required for macrophage and B cell identities. *Mol Cell* 38: 576-589

Kent WJ, Sugnet CW, Furey TS, Roskin KM, Pringle TH, Zahler AM, Haussler D (2002) The human genome browser at UCSC. *Genome Res* 12: 996-1006

Langmead B, Salzberg SL (2012) Fast gapped-read alignment with Bowtie 2. *Nat Methods* 9: 357-359

Lareau CA, Aryee MJ (2018) hichipper: a preprocessing pipeline for calling DNA loops from HiChIP data. *Nat Methods* 15: 155-156

Li H, Handsaker B, Wysoker A, Fennell T, Ruan J, Homer N, Marth G, Abecasis G, Durbin R, Genome Project Data Processing S (2009) The Sequence Alignment/Map format and SAMtools. *Bioinformatics* 25: 2078-2079

Liu Y, Sun J, Zhao M (2017) ONGene: A literature-based database for human oncogenes. *J Genet Genomics* 44: 119-121

Liu Y, Wu Z, Zhou J, Ramadurai DKA, Mortenson KL, Aguilera-Jimenez E, Yan Y, Yang X, Taylor AM, Varley KE *et al* (2021) A predominant enhancer co-amplified with the SOX2 oncogene is necessary and sufficient for its expression in squamous cancer. *Nat Commun* 12: 7139

Love MI, Huber W, Anders S (2014) Moderated estimation of fold change and dispersion for RNA-seq data with DESeq2. *Genome Biol* 15: 550

Meyers RM, Bryan JG, McFarland JM, Weir BA, Sizemore AE, Xu H, Dharia NV, Montgomery PG, Cowley GS, Pantel S *et al* (2017) Computational correction of copy number effect improves specificity of CRISPR-Cas9 essentiality screens in cancer cells. *Nat Genet* 49: 1779-1784

Quinlan AR, Hall IM (2010) BEDTools: a flexible suite of utilities for comparing genomic features. *Bioinformatics* 26: 841-842

Ramirez F, Ryan DP, Gruning B, Bhardwaj V, Kilpert F, Richter AS, Heyne S, Dundar F, Manke T (2016) deepTools2: a next generation web server for deep-sequencing data analysis. *Nucleic Acids Res* 44: W160-165

Robinson JT, Thorvaldsdottir H, Turner D, Mesirov JP (2023) igv.js: an embeddable JavaScript implementation of the Integrative Genomics Viewer (IGV). *Bioinformatics* 39

Sahu B, Hartonen T, Pihlajamaa P, Wei B, Dave K, Zhu F, Kaasinen E, Lidschreiber K, Lidschreiber M, Daub CO *et al* (2022) Sequence determinants of human gene regulatory elements. *Nat Genet* 54: 283-294

Servant N, Varoquaux N, Lajoie BR, Viara E, Chen CJ, Vert JP, Heard E, Dekker J, Barillot E (2015) HiC-Pro: an optimized and flexible pipeline for Hi-C data processing. *Genome Biol* 16: 259

Sherman BT, Hao M, Qiu J, Jiao X, Baseler MW, Lane HC, Imamichi T, Chang W (2022) DAVID: a web server for

functional enrichment analysis and functional annotation of gene lists (2021 update). *Nucleic Acids Res* 50: W216-W221
Sondka Z, Bamford S, Cole CG, Ward SA, Dunham I, Forbes SA (2018) The COSMIC Cancer Gene Census: describing genetic dysfunction across all human cancers. *Nat Rev Cancer* 18: 696-705
Tang Z, Kang B, Li C, Chen T, Zhang Z (2019) GEPIA2: an enhanced web server for large-scale expression profiling and interactive analysis. *Nucleic Acids Res* 47: W556-W560
Thomas-Chollier M, Hufton A, Heinig M, O'Keeffe S, Masri NE, Roider HG, Manke T, Vingron M (2011) Transcription factor binding predictions using TRAP for the analysis of ChIP-seq data and regulatory SNPs. *Nat Protoc* 6: 1860-1869
Tsherniak A, Vazquez F, Montgomery PG, Weir BA, Kryukov G, Cowley GS, Gill S, Harrington WF, Pantel S, Krill-Burger JM *et al* (2017) Defining a Cancer Dependency Map. *Cell* 170: 564-576 e516
Wang Q, Li M, Wu T, Zhan L, Li L, Chen M, Xie W, Xie Z, Hu E, Xu S *et al* (2022) Exploring Epigenomic Datasets by ChIPseeker. *Curr Protoc* 2: e585
Whyte WA, Orlando DA, Hnisz D, Abraham BJ, Lin CY, Kagey MH, Rahl PB, Lee TI, Young RA (2013) Master transcription factors and mediator establish super-enhancers at key cell identity genes. *Cell* 153: 307-319
Yu G, Wang LG, He QY (2015) ChIPseeker: an R/Bioconductor package for ChIP peak annotation, comparison and visualization. *Bioinformatics* 31: 2382-2383
Zhang Y, Liu T, Meyer CA, Eeckhoute J, Johnson DS, Bernstein BE, Nusbaum C, Myers RM, Brown M, Li W *et al* (2008) Model-based analysis of ChIP-Seq (MACS). *Genome Biol* 9: R137

Appendix Table S1

Additional plasmids with clone used in this study.

Plasmids for luciferase assay	Reference or Source	Identifier or Catalog Number
1st_EBF	This study	N/A
ETS	This study	N/A
ASCL	This study	N/A
2nd_SOX	This study	N/A
SNAI	This study	N/A
HNF4	This study	N/A

Plasmids for CRISPRi assay	Reference or Source	Identifier or Catalog Number
lentiGuide-NC1	Liu et al (2021)	N/A
lentiGuide-NC2	Liu et al (2021)	N/A
lentiGuide-Ctrl-1	This study	N/A
lentiGuide-Ctrl-2	This study	N/A
lentiGuide-MYB-e1-1	This study	N/A
lentiGuide-MYB-e1-2	This study	N/A
lentiGuide-MYB-e2-1	This study	N/A
lentiGuide-MYB-e2-2	This study	N/A
lentiGuide-MYB-e3-1	This study	N/A
lentiGuide-MYB-e3-2	This study	N/A
lentiGuide-MYB-e4-1	This study	N/A
lentiGuide-MYB-e4-2	This study	N/A
lentiGuide-MYB-e4-3	This study	N/A
lentiGuide-MYB-e4-4	This study	N/A
lentiGuide-MYB-e5-1	This study	N/A
lentiGuide-MYB-e5-2	This study	N/A
lentiGuide-MYB-e6-1	This study	N/A
lentiGuide-MYB-e6-2	This study	N/A
lentiGuide-MYB-e7-1	This study	N/A
lentiGuide-MYB-e7-2	This study	N/A
lentiGuide-GATA6-e1	This study	N/A
lentiGuide-GATA6-e2	This study	N/A
lentiGuide-ZFP36L2-e1	This study	N/A
lentiGuide-ZFP36L2-e2	This study	N/A

Plasmids for CRISPRa assay	Reference or Source	Identifier or Catalog Number
pXPR502-NC1-1	Liu et al (2021)	N/A
pXPR502-NC2-2	Liu et al (2022)	N/A
pXPR502-MYB-e1-2	This study	N/A
pXPR502-MYB-e2-2	This study	N/A
pXPR502-MYB-e4-1	This study	N/A
pXPR502-MYB-e4-2	This study	N/A

Plasmids for CRISPR cutting assay	Reference or Source	Identifier or Catalog Number
lentiGuide-AAVS1	Liu et al (2021)	N/A
lentiGuide-HNF4	This study	N/A

lentiGuide-1st_EBF	This study	N/A
lentiGuide-SNAI	This study	N/A
lentiGuide-ASCL	This study	N/A
lentiGuide-1st_SOX	This study	N/A

Plasmids for shRNA assay	Reference or Source	Identifier or Catalog Number
pLKO.1-shGFP	This study	N/A
pLKO.1-shMYB-3	This study	N/A
pLKO.1-shMYB-6	This study	N/A
pLKO.1-shEBF1-1	This study	N/A
pLKO.1-shEBF1-2	This study	N/A
pLKO.1-shHNF4A-1	This study	N/A
pLKO.1-shHNF4A-2	This study	N/A
pLKO.1-shHNF4G-1	This study	N/A
pLKO.1-shHNF4G-2	This study	N/A
pLKO.1-shHNF4G-3	This study	N/A

Appendix Figures

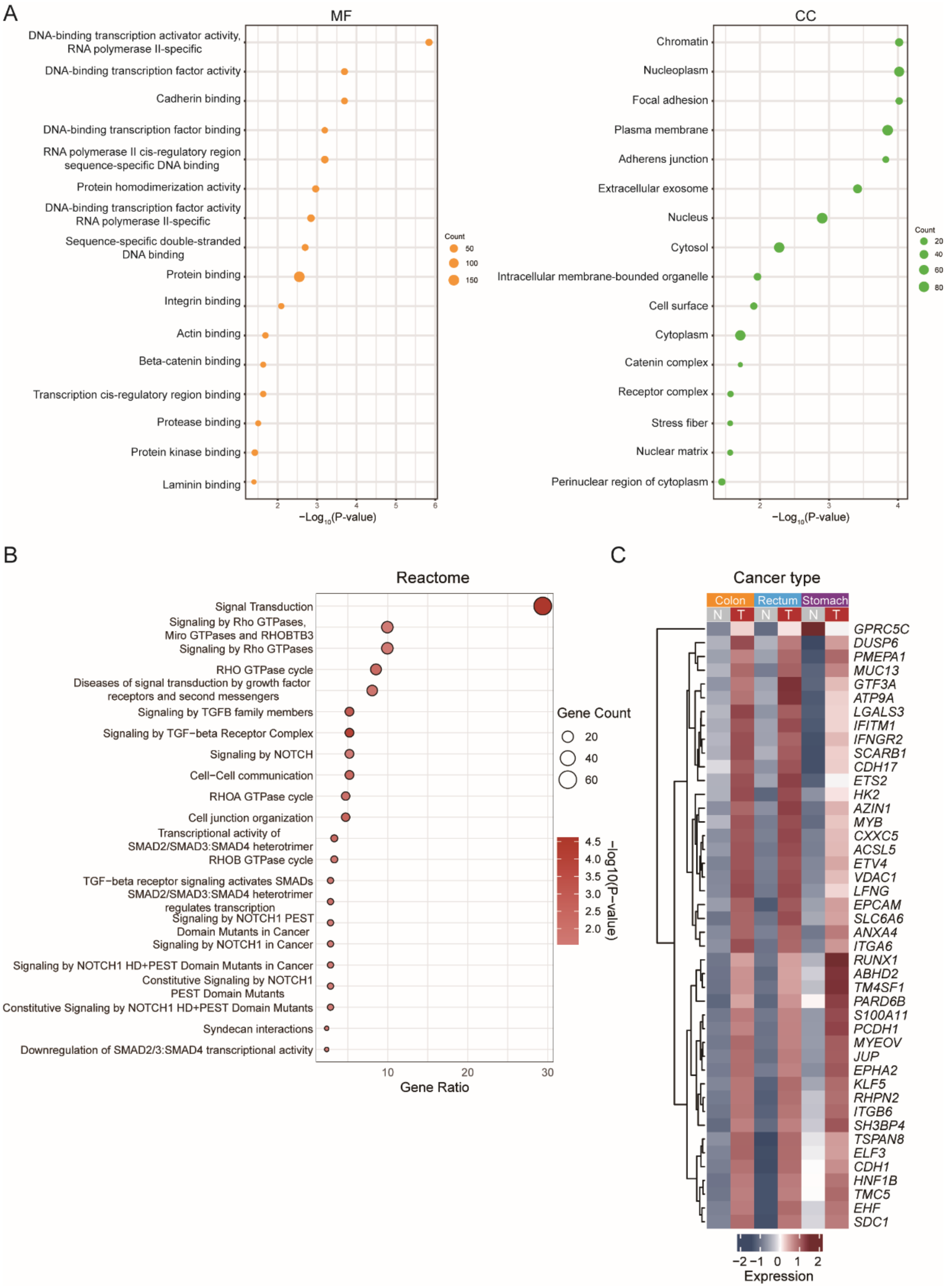

Appendix Figure S1: Enrichment and functional analysis of the common SEs nominated protein-coding genes.

A: Enrichment and functional analysis of 205 SEs nominated protein-coding genes. MF: molecular function. CC: cellular component. The *P*-value was determined by Benjamini correction method.

B: Reactome analysis for 205 SEs nominated protein-coding genes. The *P*-value was determined by Benjamini correction method.

C: The expression of 44 genes that are upregulated in tumor samples is presented in a heatmap.

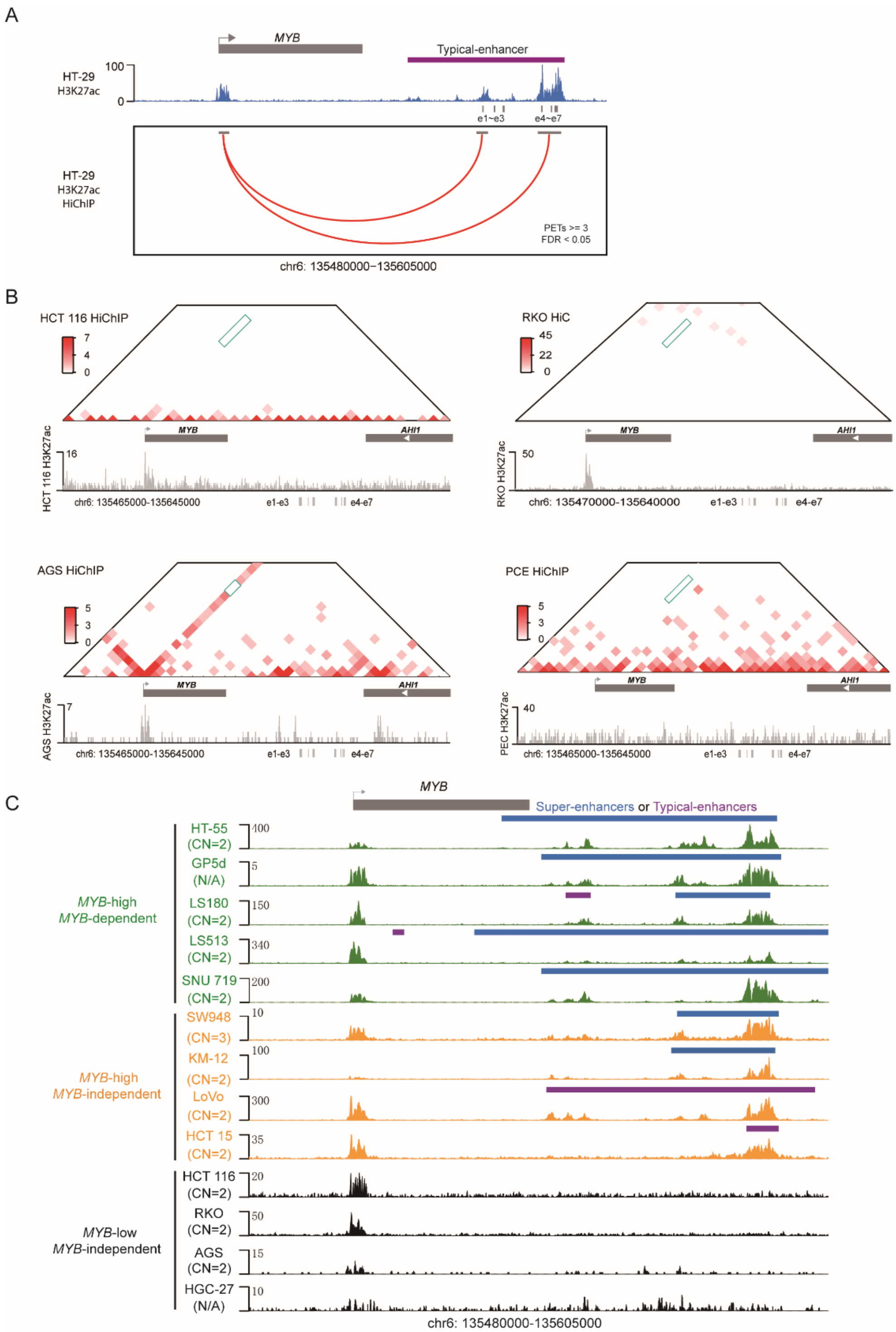

Appendix Figure S2: The chromatin landscape of the *MYB* locus in gastrointestinal adenocarcinoma.

A: Chromatin loops based on H3K27ac HiChIP data connected the *MYB* promoter to multiple distal elements with high-confidence PETs in the HT-29 cells, a *MYB*-high/-independent cell line. Note: there is no *MYB*-SE in the HT-29 cells.

B: The integration of H3K27ac ChIP-seq, HiChIP, or Hi-C data showing the three-dimensional structure of the *MYB* locus in HCT 116, RKO, AGS and PCE cells. PCE: normal human primary colonic epithelial cells. Turquoise box highlighted the absence of interaction between the *MYB* promoter and *MYB*-SE.

C: H3K27ac ChIP-seq tracks at the *MYB* locus in *MYB*-high/-dependent, *MYB*-high/-independent and *MYB*-low/-independent gastrointestinal adenocarcinoma cancer cell lines. The copy number status of *MYB* is indicated below the cell line names.

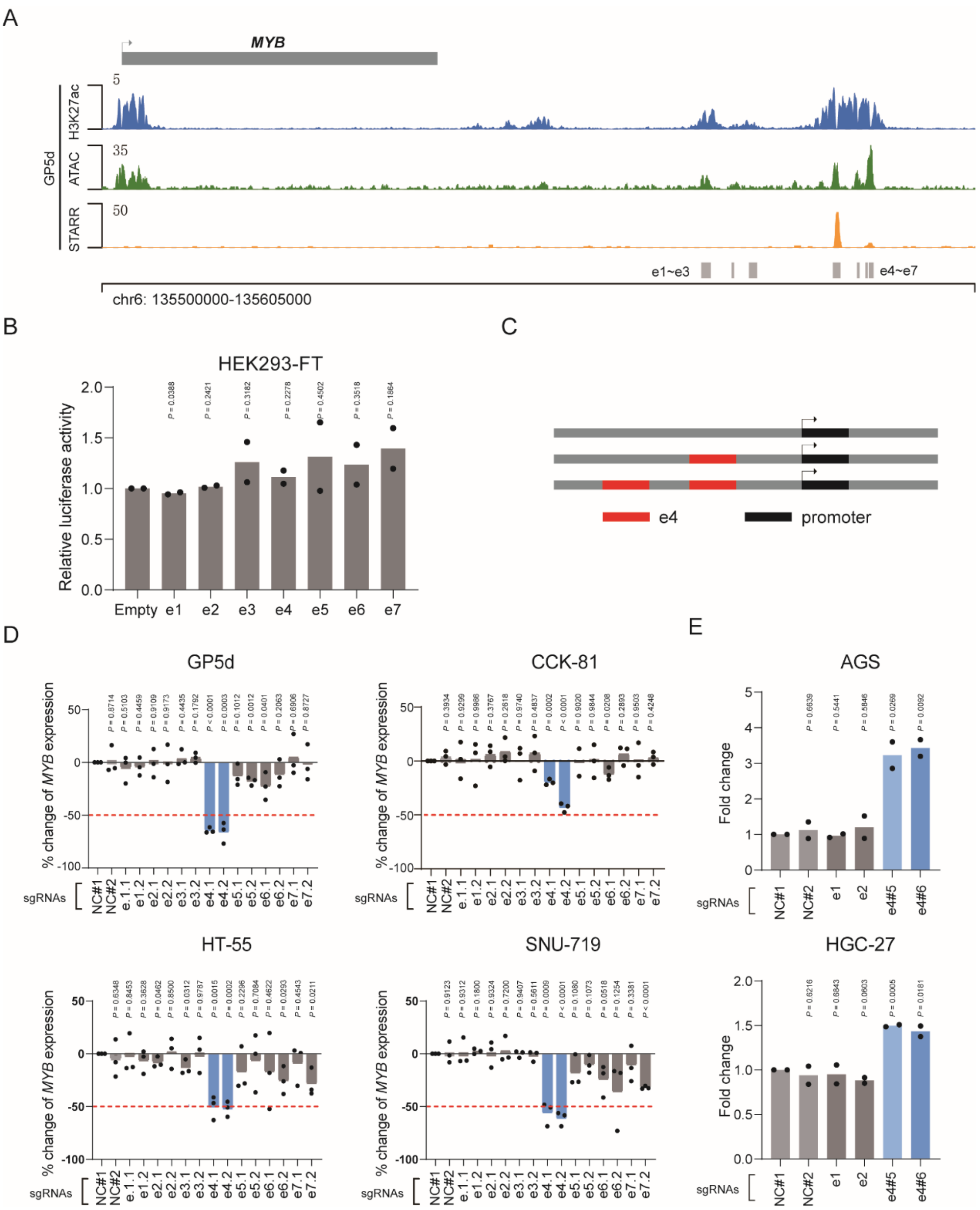

Appendix Figure S3: The enhancer e4 regulates the expression of *MYB*.

A: The H3K27ac ChIP-seq, ATAC-seq and STARR-seq tracks at the *MYB*-SE locus of in GP5d cells. Note that e4 exhibits the strongest STARR-seq signal.

B: Luciferase reporter assay measuring the activity of candidate enhancers in HEK293-FT cells. The empty pGL3-promoter vector was used as a negative control. N = 2. All values are mean ± S.D. The *P*-value was determined by two-tailed Student's *t*-test.

C: Schematic representation of e4 duplication in pGL3-promoter vector.

D: The effects of CRISPRi on the candidate enhancers regulating *MYB* expression in GP5d, CCK-81, HT-55, and SNU-719 cells. N = 3. All values are mean ± S.D. The *P*-value was determined by two-sided Student's *t*-test.

E: The effects of CRISPRa on e4 regulating *MYB* expression in two *MYB*-low/-independent cell lines AGS and HGC-27. N = 2. All values are mean ± S.D. The *P*-value was determined by two-sided Student's *t*-test.

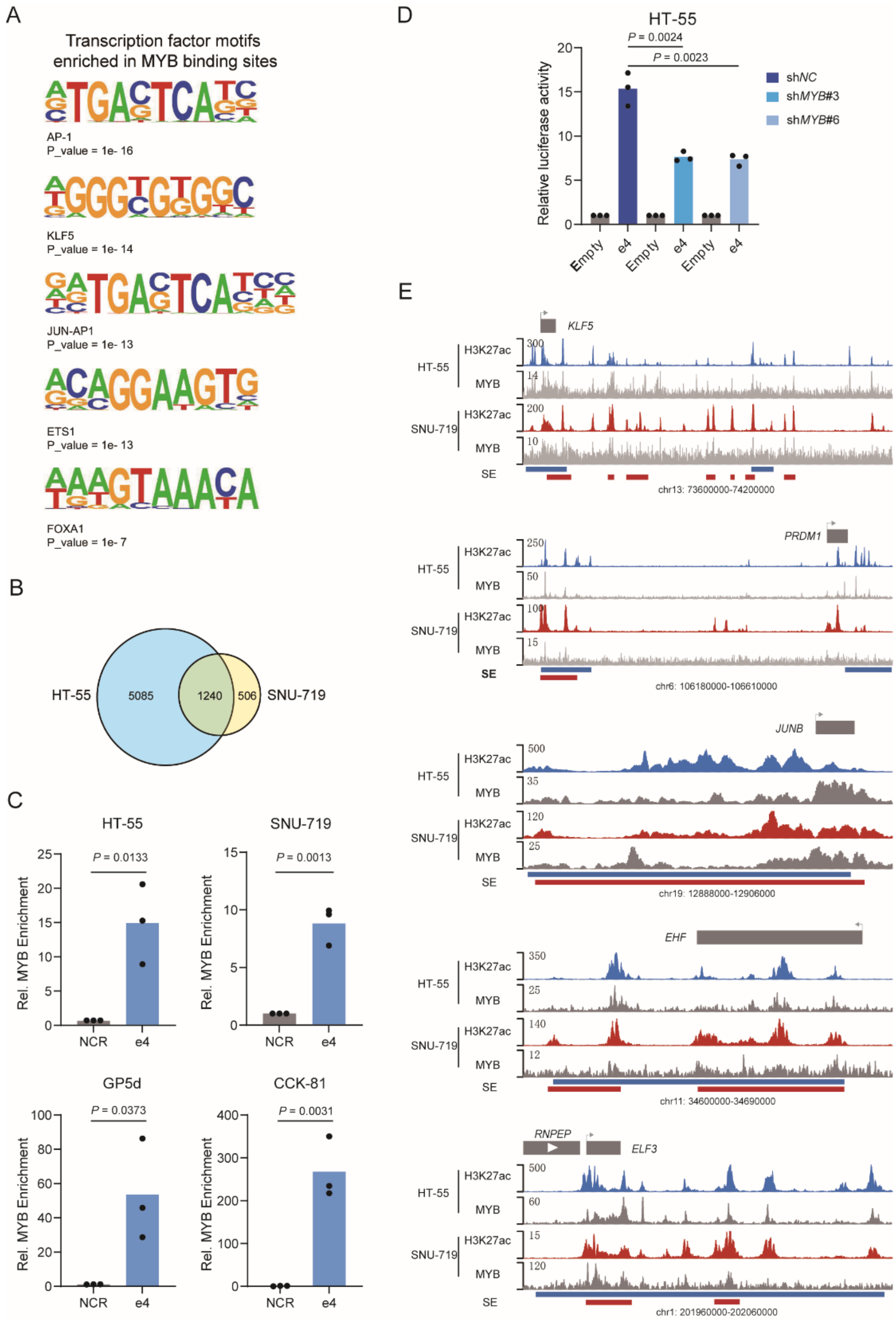

Appendix Figure S4：Genome-wide identification of MYB binding sites in HT-55 and SNU-719 cells.

A: DNA binding motifs identified using HOMER derived from MYB ChIP-seq in HT-55 and SNU-719 cells.

B: Venn diagram showing the overlap of genes with MYB binding bound by MYB in HT-55 and SNU-719 cells.

C: ChIP-qPCR analysis showing the enrichment of MYB at e4 in HT-55 and SNU-719, GP5d and CCK-81 cells. NCR: Negative control region. N = 3. All values are mean ± S.D. The *P*-value was determined by two-sided Student's *t*-test.

D: Luciferase reporter assay measuring the activity of e4 upon *MYB* knockdown in HT-55 cells. N = 3. All values are mean ± S.D. The *P*-value was determined by two-sided Student's *t*-test.

E: The MYB ChIP-seq tracks of MYB targeted cancer-related genes in HT-55 and SNU-719 cells.

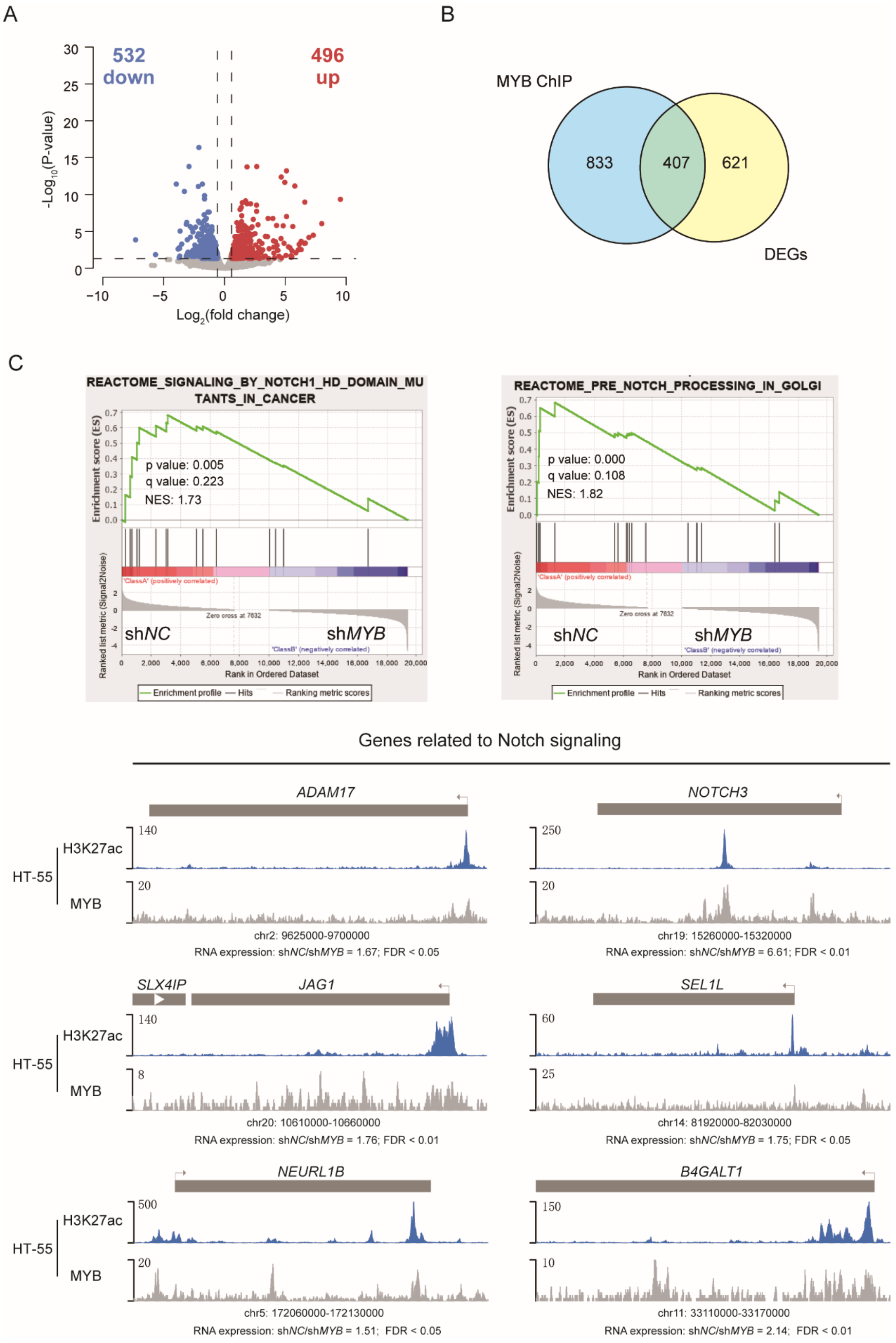

Appendix Figure S5: Identification of MYB targeted genes in HT-55 cells.

A: Volcano plot showing differentially expressed genes in HT-55 cells upon *MYB* knockdown. Red: upregulated genes upon *MYB* knockdown. Blue: downregulated genes upon *MYB* knockdown. Grey: unchanged genes.

B: Venn diagram showing genes bound and regulated by MYB.

C: GSEA analysis indicated that genes associated with Notch Signaling were downregulated upon *MYB* knockdown. NES, normalized enrichment score.
