# Supplementary material for: Systemic genome-epigenome analysis captures a lineage-specific super-enhancer for MYB in gastrointestinal adenocarcinoma
Source: Mol Syst Biol. 2025 Apr 15;21(6):696–719. doi: 10.1038/s44320-025-00098-1 (PMC12130324; doi:10.1038/s44320-025-00098-1)
Supplement: Supplementary file 2 — Table EV1 [file 44320_2025_98_MOESM2_ESM.pdf]

Table EV1: Super-enhancers nominated 205 protein-coding genes
Notes:
Interaction: genes that interact with associated SEs in both HT-55 and SNU-719 cells
Upregulation: genes that are upregulated in COREAD and STAD tumor samples compared to normal sample

Gene_nam	Type	Interactior	Upregulation
ABHD2	others	yes	yes
ABLIM1	others	yes	no
ABLIM2	others	yes	no
ACSL5	others	yes	yes
ACTN1	others	yes	no
ACTN4	oncogene	yes	no
ADAP1	others	yes	no
AFAP1	others	yes	no
AHNAK	others	yes	no
ALDH2	others	yes	no
AMN1	others	yes	no
ANXA11	others	yes	no
ANXA4	others	yes	yes
ARHGAP2€	others	yes	no
ARHGEF1C	others	yes	no
ASB9P1	others	no	no
ATP1B1	others	yes	no
ATP9A	others	yes	yes
AZIN1	others	yes	yes
BCL11A	oncogene	yes	no
BCL2L1	oncogene	yes	no
BCOR	others	yes	no
BDH1	others	yes	no
BHLHE40	others	yes	no
BMF	others	yes	no
C6orf132	others	yes	no
C9orf50	others	no	no
CASZ1	others	yes	no
CD9	others	yes	no
CDH1	oncogene	yes	yes
CDH17	oncogene	yes	yes
CEBPA	others	yes	no
CFLAR	oncogene	yes	no
CGN	others	no	no
CHN2	others	no	no
CLRN3	others	no	no
CPEB4	others	yes	no
CRIP1	others	no	no
CSRNP1	others	yes	no
CTDSP2	others	yes	no
CTNND1	others	yes	no
CUEDC1	others	yes	no
CXXC5	others	yes	yes
DEGS2	others	no	no
DUSP6	others	yes	yes

ECE1	others	no	no
EEA1	others	no	no
EGLN3	others	yes	no
EHF	others	yes	yes
EIF4B	others	no	no
ELF3	oncogene	yes	yes
EMBP1	others	no	no
EPB41L1	others	yes	no
EPCAM	oncogene	yes	yes
EPHA2	oncogene	yes	yes
ETS2	oncogene	yes	yes
ETV4	oncogene	yes	yes
FLNB	others	yes	no
FOSL2	others	yes	no
FOXP4	others	yes	no
FZD5	others	yes	no
GADD45A	others	yes	no
GATA6	oncogene	yes	no
GCOM1	others	yes	no
GDPD5	others	no	no
GNE	others	no	no
GPRC5C	others	yes	yes
GSE1	others	yes	no
GTF3A	others	yes	yes
HES1	others	yes	no
HK2	others	yes	yes
HNF1B	others	yes	yes
HS1BP3	others	no	no
ID1	oncogene	yes	no
IER2	others	yes	no
IER5	others	yes	no
IER5L	others	yes	no
IFITM1	others	yes	yes
IFNGR2	others	yes	yes
INF2	others	yes	no
INO80D	others	yes	no
IRF1	others	yes	no
ITGA6	others	yes	yes
ITGB1	others	yes	no
ITGB6	others	yes	yes
JAG1	others	yes	no
JUNB	oncogene	yes	no
JUP	oncogene	yes	yes
KALRN	others	yes	no
KCNK5	others	yes	no
KLF13	others	yes	no
KLF5	oncogene	yes	yes
KLF6	others	yes	no
KRT42P	others	no	no
KSR1	others	yes	no

LBR	others	yes	no
LFNG	others	yes	yes
LGALS3	others	yes	yes
LIPH	others	yes	no
LITAF	others	yes	no
LLGL2	others	yes	no
LMNA	others	yes	no
LRP5	oncogene	yes	no
MALAT1	oncogene	yes	no
MBNL2	others	yes	no
MCF2L	oncogene	yes	no
MEF2D	oncogene	yes	no
MIDEAS	others	yes	no
MPRIP	others	yes	no
MUC13	others	yes	yes
MYB	oncogene	yes	yes
MYEOV	others	yes	yes
MYH9	others	yes	no
MYOF	others	yes	no
NCOR2	others	yes	no
NEAT1	oncogene	yes	no
NFKBIA	others	yes	no
NHSL3	others	yes	no
NIBAN2	others	yes	no
NOTCH1	oncogene	yes	no
OGDH	others	yes	no
PARD6B	others	yes	yes
PBX1	oncogene	no	no
PCDH1	others	yes	yes
PIK3C2B	others	yes	no
PKDCC	others	yes	no
PLEC	others	yes	no
PLEKHA2	others	no	no
PLEKHG3	others	yes	no
PLS3	others	no	no
PMEPA1	others	yes	yes
POLD4	others	yes	no
POU5F1B	others	no	no
PRKCD	others	yes	no
PROC	others	yes	no
PRR15L	others	yes	no
PSMG1	others	no	no
PTP4A2	oncogene	yes	no
PTPRF	others	yes	no
PTPRJ	others	yes	no
PTTG2	oncogene	no	no
RGS3	others	yes	no
RHPN2	others	yes	yes
RIN2	others	yes	no
ROCK2	others	yes	no

RUNX1	oncogene	yes	yes
S100A11	others	yes	yes
SCARB1	others	yes	yes
SDC1	others	yes	yes
SDC4	others	yes	no
SEMA4B	others	yes	no
SERINC5	others	no	no
SH2D6	others	yes	no
SH3BP4	others	yes	yes
SH3PXD2A	others	yes	no
SHB	others	yes	no
SLC16A13	others	no	no
SLC6A6	others	yes	yes
SLC9A8	others	yes	no
SLCO2B1	others	yes	no
SMAD3	others	yes	no
SMAD7	others	yes	no
SNORA14E	others	yes	no
SNORD17	others	no	no
SNX29P2	others	no	no
SOWAHC	others	yes	no
SP1	others	no	no
SPSB1	others	yes	no
SREBF1	oncogene	yes	no
ST3GAL2	others	yes	no
STAMBPL1	others	yes	no
STARD10	others	yes	no
SUMO1P1	others	no	no
SYNJ2	others	yes	no
SYTL2	others	yes	no
TBC1D14	others	yes	no
TBL1XR1	oncogene	no	no
TGIF1	others	no	no
TGM2	others	no	no
TM4SF1	others	yes	yes
TMC5	others	yes	yes
TMED10	others	no	no
TMEM105	others	no	no
TMPRSS2	oncogene	yes	no
TMSB4X	oncogene	yes	no
TNRC18	others	yes	no
TNS3	others	yes	no
TOB1	others	yes	no
TRIB1	oncogene	yes	no
TRIM2	others	yes	no
TSEN2	others	yes	no
TSPAN8	others	yes	yes
UBC	others	yes	no
VDAC1	others	yes	yes
XBP1	oncogene	yes	no

ZBTB38	others	yes	no
ZBTB7B	others	no	no
ZC3H4	others	yes	no
ZFP36	others	yes	no
ZFP36L2	others	yes	no
ZMYND8	others	yes	no
ZNF703	oncogene	no	no
ZNF706	others	yes	no
ZNF750	others	no	no
ZNRF3	others	yes	no

s.
