# Supplementary material for: Systemic genome-epigenome analysis captures a lineage-specific super-enhancer for MYB in gastrointestinal adenocarcinoma
Source: Mol Syst Biol. 2025 Apr 15;21(6):696–719. doi: 10.1038/s44320-025-00098-1 (PMC12130324; doi:10.1038/s44320-025-00098-1)
Supplement: Supplementary file 3 — Table EV2 [file 44320_2025_98_MOESM3_ESM.pdf]

Table EV2: Publicly available data used in this study

HiChIP and Hi-C data

Sample information	GEO accession number
HT-55_H3K27ac HiChIP	GSM4447216
GP5d_H3K27ac HiChIP	GSM6841219
HCT 116_H3K27ac HiChIP	GSM4053397
LS513 H3K27ac HiChIP	GSM4053398
SW620_H3K27ac HiChIP	GSM4053400
SNU-719_H3K27ac HiChIP	GSM7679532
AGS_H3K27ac HiChIP	GSE235431
RKO_Hi-C	GSM3930292
HT-29_H3K27ac HiChIP	GSM7117686
Human Primary Colonic Epithelial Cells_H3K27ac HiChIP	GSM5680725

The H3K27ac ChIP-seq

Sample information	GEO accession number
MCF-7_H3K27ac	GSM2483408
MDA-MB-231_H3K27ac	GSM1342629
LNCaP_H3K27ac	GSM1902615
VCaP_H3K27ac	GSM2827606
A549_H3K27ac	GSM5330933
NCI-H358_H3K27ac	GSM1635575
Ishikawa_H3K27ac	GSM1635579
Hep-G2_H3K27ac	GSM3495143
5637_H3K27ac	GSM5821795
AsPC1_H3K27ac	GSM3178667
MOLM-13_H3K27ac	GSM4796338
THP-1_H3K27ac	GSM5908232
GSU_H3K27ac	GSM2356648
SNU-719_H3K27ac	GSM3991522
GP5d_H3K27ac	GSM5454417
SW620_H3K27ac	GSM4060933
LoVo_H3K27ac	GSM3592807
HCTc15_H3K27ac	GSM4060937
RKO_H3K27ac	GSM4060939
HCT 116_H3K27ac	GSM1890730
V576_H3K27ac	GSM2058044
V784_H3K27ac	GSM2058046
V457_H3K27ac	GSM2058041
V481_H3K27ac	GSM2058042
ESO-26_H3K27ac	GSM3886296
SK-GT-4_H3K27ac	GSM3886317
KYAE-1_H3K27ac	GSM3886306
OE19_H3K27ac	GSM3886313
OE33_H3K27ac	GSM3886315
JH-EsoAd1_H3K27ac	GSM3886304
HGC-27_H3K27ac	GSM5951765
LS513_H3K27ac	GSM4053398
LS180_H3K27ac	GSM1890754
AGS_H3K27ac	GSM2356641

FLO-1_H3K27ac	GSM3886302
KM12	GSM4271273
SW948	GSM4060931
T016	GSM4061148
T017	GSM4061151
T990275	GSM3315814
T20020720	GSM3356984

Other

Sample information	GEO accession number
GP5D_STARR	GSM5454434
GP5D_ATAC	GSM5454363
GP5D_CTCF	GSM5454415
DLD1_CTCF	GSM4238557
SNU719_CTCF	GSM7471656
GP5D_HNF4A	GSM5454423
L2-3_H3K27ac	GSE232459
3346_H3K27ac	
L2-3_RNA-seq	
3346_RNA-seq	
