# Supplementary material for: Systemic genome-epigenome analysis captures a lineage-specific super-enhancer for MYB in gastrointestinal adenocarcinoma
Source: Mol Syst Biol. 2025 Apr 15;21(6):696–719. doi: 10.1038/s44320-025-00098-1 (PMC12130324; doi:10.1038/s44320-025-00098-1)
Supplement: Supplementary file 4 — Table EV3 [file 44320_2025_98_MOESM4_ESM.pdf]

Table EV3: The 1240 target genes closest to the MYB binding site both in HT-55 SNU-719 cells

DDX11L1	*CACNA1E*	*ATL3*	*PDX1*	*ABHD17C*	*SLC7A5*	*ARHGDIA*
WASH7P	*LAMC2*	*MACROD1*	*BRCA2*	*MIR549A*	*BANP*	*NOTUM*
OR4F29	*COLGALT2*	*STIP1*	*RFC3*	*ABHD2*	*IL17C*	*FASN*
RNF223	*INAVA*	*FKBP2*	*PROSER1*	*ZNF710*	*PIEZO1*	*CCDC57*
LINC01342	*PHLDA3*	*CDC42BPG*	*SUCLA2*	*IDH2*	*ZNF778*	*FOXK2*
NADK	*BTG2*	*SYVN1*	*CAB39L*	*SEMA4B*	*CDK10*	*WDR45B*
ARHGEF16	*ELK4*	*MALAT1*	*DACH1*	*MEF2A*	*ZNF276*	*FN3KRP*
KLHL21	*CD46*	*OVOL1*	*KLF5*	*ASB7*	*AFG3L1P*	*B3GNTL1*
CAMTA1	*SERTAD4*	*EIF1AD*	*KLF12*	*SNRPA1*	*PRDM7*	*RPL23AP87*
ENO1	*PPP2R5A*	*MRPL11*	*LMO7*	*FAM234A*	*RPH3AL*	*MGC70870*
KIAA2013	*ATF3*	*DPP3*	*NDFIP2*	*LINC00235*	*GLOD4*	*ROCK1P1*
TMEM51	*LINC00538*	*RBM14-RBM4*	*DCT*	*MIR5587*	*BHLHA9*	*DLGAP1-AS1*
CTRC	*H3P6*	*RBM4B*	*STK24*	*RAB40C*	*MNT*	*LOC644669*
FBLIM1	*PSEN2*	*C11orf80*	*UBAC2*	*ANTKMT*	*SPNS2*	*ROCK1*
EPHA2	*RNF187*	*LRFN4*	*MYO16-AS1*	*SOX8*	*GLTPD2*	*GATA6*
CROCCP3	*DUSP5P1*	*KMT5B*	*IRS2*	*UBE2I*	*PFN1*	*CABLES1*
MIR3675	*KCNK1*	*LRP5*	*ING1*	*UNKL*	*ZNF232*	*SMAD2*
CROCC	*IRF2BP2*	*TPCN2*	*ANKRD10*	*JPT2*	*YBX2*	*ZBTB7C*
RNF186	*LINC01132*	*MYEOV*	*ATP11A*	*MAPK8IP3*	*POLR2A*	*ATP8B1-AS1*
MDS2	*SNORA14B*	*FADD*	*MCF2L-AS1*	*NUBP2*	*KDM6B*	*NEDD4L*
GALE	*NID1*	*MIR3664*	*PCID2*	*TRAF7*	*PLD6*	*SERPINB8*
DHDDS	*GPR137B*	*GDPD4*	*LAMP1*	*ECI1*	*TOM1L2*	*CYB5A*
HMGN2	*ACTN2*	*PANX1*	*TMCO3*	*ZG16B*	*GRAP*	*SALL3*
SFN	*FH*	*MRE11*	*TFDP1*	*PRSS22*	*GRAPL*	*MISP*
KDF1	*ZNF695*	*ENDOD1*	*CHAMP1*	*BICDL2*	*EPN2*	*MED16*
SYTL1	*NLRP3*	*BIRC3*	*POTEM*	*ZNF263*	*MTRNR2L1*	*ARHGAP45*
IFI6	*PGBD2*	*BUD13*	*HNRNPC*	*TRAP1*	*MIR4522*	*STK11*
FAM76A	*GTPBP4*	*TREH*	*HOMEZ*	*ADCY9*	*TNFAIP1*	*CIRBP*
RNU11	*PFKFB3*	*BCL9L*	*ARHGAP5-AS1*	*SRL*	*TRAF4*	*ADAMTSL5*
SERINC2	*MIR3155B*	*USP2-AS1*	*EGLN3*	*LINC01569*	*CORO6*	*TCF3*
KIAA1522	*ST8SIA6-AS1*	*ST14*	*EAPP*	*DNAJA3*	*PIGW*	*DOT1L*
STK40	*KIAA1217*	*WASH8P*	*PPP2R3C*	*UBALD1*	*ARHGAP23*	*GADD45B*
BMP8B	*ZNF438*	*PRMT8*	*SSTR1*	*NUDT16L1*	*MLLT6*	*ZNF77*
TRIT1	*NRP1*	*ZNF384*	*FBXO33*	*PPL*	*GRB7*	*FZR1*
HIVEP3	*LINC00999*	*MIR200C*	*BMP4*	*USP7*	*THRA*	*YJU2*
FOXJ3	*LOC441666*	*MANSC1*	*GMFB*	*SNN*	*KRT42P*	*SH3GL1*
KIF2C	*EGR2*	*APOLD1*	*L3HYPDH*	*PDXDC1*	*EIF1*	*DPP9*
PLK3	*HKDC1*	*DDX47*	*PRKCH*	*MIR3180-3*	*JUP*	*UHRF1*
MAST2	*PALD1*	*RPL13AP20*	*PLEKHG3*	*COQ7*	*CCDC200*	*DUS3L*
PIK3R3	*PCBD1*	*ATF7IP*	*LOC100506321*	*VPS35L*	*LINC00910*	*KHSRP*
FOXD2	*MYOZ1*	*ITPR2*	*PLEK2*	*ERI2*	*MAP3K14*	*SH2D3A*
TXNDC12	*FUT11*	*BICD1*	*ZFP36L1*	*LYRM1*	*SP2*	*ARHGEF18*
LRP8	*VCL*	*ALG10*	*EXD2*	*SMG1P3*	*PRR15L*	*PRR36*
MRPL37	*VDAC2*	*SCAF11*	*MIDEAS*	*POLR3E*	*NFE2L1*	*DNMT1*
CYP2J2	*LIPK*	*SLC38A2*	*LINC01220*	*SULT1A1*	*SPOP*	*DNM2*
GADD45A	*EXOC6*	*BCDIN3D*	*ALKBH1*	*RABEP2*	*TOB1*	*TMED1*
SH3GLB1	*CYP2C8*	*DIP2B*	*ADCK1*	*MAZ*	*MMD*	*TIMM29*
LRRC8B	*SLC25A28*	*ATF1*	*GTF2A1*	*TBC1D10B*	*AKAP1*	*KANK2*
GFI1	*KAZALD1*	*KRT78*	*TTC8*	*PRR14*	*SRSF1*	*DOCK6*
MFSD14A	*NOLC1*	*KRT8*	*PPP4R3A*	*SETD1A*	*TEX14*	*ZNF491*
AKNAD1	*FBXL15*	*SP1*	*CATSPERB*	*ZNF668*	*TRIM37*	*ZNF443*
DENND2D	*NT5C2*	*RAB5B*	*ITPK1-AS1*	*RUSF1*	*CLTC*	*TRIR*
AP4B1	*STN1*	*CAND1*	*CLMN*	*SLC6A10P*	*MIR21*	*HOOK2*
CD58	*MXI1*	*DYRK2*	*PAPOLA*	*LOC390705*	*USP32*	*JUNB*

SRGAP2D	*TCF7L2*	*MDM2*	*LINC02914*	*LINC00273*	*CYB561*	*SYCE2*
EMBP1	*ABLIM1*	*YEATS4*	*WDR25*	*ANKRD26P1*	*PLEKHM1P1*	*ADGRL1*
ANKRD20A12P	*GRK5*	*TMEM19*	*LINC00239*	*AKTIP*	*AMZ2P1*	*AKAP8*
NA	*RGS10*	*PHLDA1*	*DYNC1H1*	*IRX5*	*LINC00673*	*AP1M1*
NBPF10	*WDR11*	*EEA1*	*ANKRD9*	*PLLP*	*JPT1*	*MRPL34*
LINC01138	*TACC2*	*UBE2N*	*KIF26A*	*CCDC102A*	*GRB2*	*MAP1S*
NBPF8	*PLEKHA1*	*CDK17*	*INF2*	*FHOD1*	*LLGL2*	*B3GNT3*
LSP1P5	*PSTK*	*TTC41P*	*AKT1*	*CTCF*	*ITGB4*	*RPL18A*
LINC02591	*DUX4L3*	*MMAB*	*CEP170B*	*DUS2*	*H3-3B*	*PDE4C*
LINC00623	*SCGB1C1*	*FAM222A*	*GPR132*	*ESRP2*	*TRIM65*	*GDF15*
MCL1	*B4GALNT4*	*TPCN1*	*CHEK2P2*	*SLC7A6*	*ACOX1*	*ELL*
ZNF687	*SIGIRR*	*KSR2*	*MIR4715*	*HAS3*	*MXRA7*	*KXD1*
CGN	*MIR210HG*	*CIT*	*CHRFAM7A*	*TERF2*	*SRSF2*	*GATAD2A*
TUFT1	*TMEM80*	*BICDL1*	*LPCAT4*	*NQO1*	*SEPTIN9*	*PBX4*
C2CD4D	*AP2A2*	*PXN-AS1*	*C15orf62*	*PDXDC2P-NPIPB14P*	*C17orf99*	*HAVCR1P1*
S100A11	*MUC6*	*SIRT4*	*SPINT1*	*AARS1*	*AFMID*	*LINC00662*
S100A2	*SYT8*	*MLEC*	*CHAC1*	*VAC14*	*PGS1*	*RHPN2*
PMVK	*MIR4686*	*HPD*	*CHP1*	*PMFBP1*	*USP36*	*LRP3*
ZBTB7B	*KCNQ1*	*MLXIP*	*FRMD5*	*FA2H*	*ENPP7*	*KCTD15*
YY1AP1	*OR7E12P*	*RSRC2*	*SHF*	*ZFP1*	*CBX8*	*GRAMD1A*
F11R	*PPFIBP2*	*NCOR2*	*USP3*	*GABARAPL2*	*CBX4*	*LSR*
TSTD1	*EIF4G2*	*MIR5188*	*PPIB*	*SDR42E1*	*TBC1D16*	*RBM42*
FCGR2A	*ZBED5-AS1*	*SFSWAP*	*PARP16*	*OSGIN1*	*NPTX1*	*THAP8*
NUF2	*TEAD1*	*PGAM5*	*SMAD6*	*ATP2C2*	*RPTOR*	*ZNF875*
PBX1	*OR4C46*	*ANHX*	*DRAIC*	*COTL1*	*SLC38A10*	*SIPA1L3*
MGST3	*TNKS1BP1*	*IFT88*	*PKM*	*GSE1*	*TMEM105*	*DPF1*
GPA33	*LPXN*	*MICU2*	*PPCDC*	*IRF8*	*BAHCC1*	*SPINT2*
CREG1	*MS4A7*	*BASP1P1*	*MAN2C1*	*C16orf95*	*ACTG1*	*C19orf33*
RABGAP1L	*SYT7*	*GTF3A*	*MINAR1*	*KLHDC4*	*MCRIP1*	*CATSPERG*
JAK2	*KDM4C*	*LURAP1L*	*CAAP1*	*PTENP1*	*MSMP*	*RNF38*
LOC100133920	*FOXD4L5*	*CBWD3*	*PIP5K1B*	*TJP2*	*ZFAND5*	*OSTF1*
CTSV	*NR4A3*	*RAD23B*	*ACTL7B*	*AKNA*	*GOLGA1*	*RABEPK*
SETX	*GTF3C4*	*RALGDS*	*PPP1R26*	*AGPAT2*	*SNHG7*	*TMEM141*
DUX4	*TBL1X*	*IL1RAPL1*	*FTSJ1*	*OTUD5*	*BMP15*	*ZXDA*
CBWD6	*ZNF782*	*PRRC2B*	*LOC100507412*	*IL9R*	*GYG2P1*	*SPRY3*
ZSCAN25	*AP4M1*	*PILRB*	*ZCWPW1*	*PPP1R35*	*FBXO24*	*SLC12A9*
FEZF1-AS1	*METTL2B*	*ATP6V0A4*	*LUC7L2*	*KDM7A*	*KDM7A-DT*	*PRSS3P2*
RHEB	*PRKAG2*	*LINC01003*	*SHH*	*PTPRN2-AS1*	*MIR595*	*PTPRN2*
FAM86B3P	*PRAG1*	*MFHAS1*	*PSD3*	*NPM2*	*DPYSL2*	*LINC00589*
CHD7	*JPH1*	*REXO1L2P*	*REXO1L1P*	*YWHAZ*	*FLJ42969*	*KLF10*
MIR30D	*TRAPPC9*	*CHRAC1*	*TSNARE1*	*LINC02904*	*ZNF696*	*SCRIB*
PRXL2C	*IER5L*	*LOC283788*	*FLNA*	*BCLAF1*	*TIAM2*	*ARID1B*
PSMD8	*ETAA1*	*BCL2L1*	*CCDC12*	*ARHGEF38*	*GTF2IRD1*	*SPIN4*
ACTN4	*APLF*	*C20orf144*	*PTPN23*	*ZGRF1*	*GTF2I*	*CXCR3*
HNRNPL	*SNORA36C*	*ZNF341*	*SMARCC1*	*USP53*	*NSUN5P1*	*FGF16*
ZFP36	*MXD1*	*AHCY*	*ZNF589*	*MGST2*	*POM121C*	*ATRX*
SERTAD1	*FAM136A*	*DYNLRB1*	*NDUFAF3*	*SMAD1*	*HSPB1*	*PLS3*
CYP2S1	*TET3*	*PIGU*	*USP4*	*FBXW7*	*SSC4D*	*RASA4*
AXL	*MTHFD2*	*RBM39*	*MST1R*	*CBR4*	*ZP3*	*LHFPL3-AS2*
CEACAM6	*RTKN*	*NORAD*	*DCAF1*	*GALNT7*	*DTX2*	*KMT2E-AS1*
ARHGEF1	*HK2*	*CHD6*	*ACY1*	*TRAPPC11*	*PTPN12*	*SYPL1*
RABAC1	*CTNNA2*	*SLC35C2*	*TLR9*	*FRG1*	*PHTF2*	*IFRD1*
ERF	*TMSB10*	*SULF2*	*APPL1*	*DUX4L8*	*SEMA3A*	*MUC12*
ZNF428	*SH2D6*	*LINC00494*	*DNAH12*	*LINC01667*	*STEAP2*	*SERPINE1*
SMG9	*PARTICL*	*ARFGEF2*	*LRIG1*	*PLEKHG4B*	*FAM133B*	*ZNHIT1*
ZNF112	*IMMT*	*SNAI1*	*SHQ1*	*AHRR*	*TECPR1*	*MYL10*

POLR1G	*RNF103*	*PEDS1-UBE2V1*	*EBLN2*	*SLC9A3*	*PDAP1*	*CUX1*
ERCC1	*CYTOR*	*LINC01270*	*LINC00960*	*CEP72-DT*	*FAM27B*	*ARHGEF5*
FOSB	*EIF2AK3*	*PARD6B*	*LINC01205*	*SLC12A7*	*CDC14B*	*EZH2*
VASP	*MIR4436A*	*BCAS4*	*C3orf52*	*TERT*	*PHYHD1*	*LOC155060*
OPA3	*LSP1P4*	*ZNF217*	*KALRN*	*MIR4457*	*FAM230C*	*REPIN1*
DMPK	*ACTR3BP2*	*SUMO1P1*	*PLXNA1*	*LINC02111*	*THOC2*	*ZNF775*
IRF2BP1	*FAHD2CP*	*CYP24A1*	*MGLL*	*EMB*	*FAM3C*	*ESYT2*
IGFL4	*ASTL*	*SPO11*	*BFSP2*	*ANKRD55*	*POLR2J3*	*DYNC2I1*
AP2S1	*STARD7*	*ATP5F1E*	*SOX14*	*ZSWIM6*	*ABCB8*	*LOC389831*
ZC3H4	*ANKRD36*	*CDH26*	*TM4SF18*	*CD180*	*AGPAT5*	*MIR596*
MIR3190	*MGAT4A*	*LSM14B*	*TM4SF1*	*PIK3R1*	*ST18*	*KBTBD11*
BICRA	*C2orf15*	*LAMA5*	*TSC22D2*	*GUSBP17*	*PVT1*	*EIF4EBP1*
ODAD1	*FHL2*	*PPDPF*	*SIAH2*	*ANKRD31*	*FRG1HP*	*GOLGA7*
EMP3	*MIR4265*	*UCKL1*	*C3orf33*	*F2RL1*	*MRPS17*	*KAT6A*
SULT2B1	*BCL2L11*	*C20orf204*	*TIPARP*	*HOMER1*	*LOC650226*	*POTEA*
FAM83E	*MIR4435-2HG*	*MIR3648-1*	*LEKR1*	*SERINC5*	*ZNF733P*	*LINC00293*
FUT1	*TMEM87B*	*TPTE*	*LINC00880*	*MTRNR2L2*	*ZNF92*	*AZIN1*
SLC17A7	*ZC3H6*	*BAGE3*	*CCNL1*	*MIR9-2*	*LINC03006*	*EBAG9*
NOSIP	*POLR1B*	*EVA1C*	*LRRC77P*	*CCDC112*	*RABGEF1*	*SNTB1*
SCAF1	*SLC20A1*	*IFNGR2*	*GOLIM4*	*SEMA6A*	*TMEM248*	*FER1L6*
IRF3	*RABL2A*	*RUNX1*	*MYNN*	*ZNF608*	*SBDS*	*POU5F1B*
MYH14	*LINC01191*	*CBR3*	*GPR160*	*SLC12A2-DT*	*NSUN5P2*	*PUF60*
JOSD2	*TSN*	*TTC3*	*PLD1*	*MIR4460*	*ABHD11*	*TMEM242*
C19orf48	*CNTNAP5*	*VPS26C*	*FNDC3B*	*RAPGEF6*	*CLDN4*	*ZDHHC14*
RPS9	*IMP4*	*ETS2*	*ECT2*	*JADE2*	*CLIP2*	*SNX9*
RDH13	*ANKRD30BL*	*PRDM15*	*TBL1XR1*	*C5orf24*	*POLD2*	*SYNJ2*
EPS8L1	*MIR663B*	*PKNOX1*	*USP13*	*KIF20A*	*NUDCD3*	*SMOC2*
KMT5C	*WDSUB1*	*CRYAA*	*LINC01994*	*EGR1*	*DDX56*	*CYC1*
UBE2S	*PSMD14*	*LINC00319*	*MCCC1*	*CYSTM1*	*OGDH*	*PUF60*
EPN1	*STK39*	*PDXK*	*POLR2H*	*ANKHD1-EIF4EBP3*	*ZMIZ2*	*CYC1*
SRSF10	*DLX2*	*ICOSLG*	*EPHB3*	*APBB3*	*PPIA*	*SLC39A4*
HYDIN2	*MAP3K20*	*CCT8L2*	*LIPH*	*CSNK1A1*	*H2AZ2*	*ZNF252P*
FAM110C	*CDCA7*	*USP18*	*BCL6*	*DBN1*	*LINC00525*	*SLC1A1*
SH3YL1	*WIPF1*	*GGT3P*	*TPRG1-AS2*	*DDX41*	*UPP1*	*SMOC2*
TMEM18	*FSIP2*	*DGCR6*	*CLDN1*	*GMDS-DT*	*COBL*	*AZIN1*
TPO	*ZC3H15*	*ZNF74*	*DPPA2P3*	*SERPINB1*	*LANCL2*	*EBAG9*
SOX11	*NAB1*	*YDJC*	*LINC02026*	*RIPK1*	*VOPP1*	*SNTB1*
RNF144A	*STAT1*	*VPREB1*	*HES1*	*NRN1*	*GNA12*	*FER1L6*
ID2	*CFLAR-AS1*	*KIAA1671-AS1*	*LINC01968*	*LY86*	*AP5Z1*	*POU5F1B*
ASAP2	*FAM117B*	*LIMK2*	*ACAP2*	*DSP*	*TNRC18*	*SYNJ2*
KLF11	*RAPH1*	*PISD*	*MIR5692C1*	*CDKAL1*	*ACTB*	*EIF4EBP1*
RRM2	*IDH1*	*CARD10*	*SDHAP2*	*H4C8*	*EIF2AK1*	*GOLGA7*
ODC1	*TTLL4*	*PICK1*	*PIGX*	*LINC01623*	*USP42*	*KAT6A*
LAPTM4A	*ABCB6*	*PDGFB*	*MELTF-AS1*	*PPP1R18*	*ICA1*	*POTEA*
SDC1	*SPEG*	*RPS19BP1*	*DLG1*	*FLOT1*	*ARL4A*	*LINC00293*
TP53I3	*AP1S3*	*OGFRP1*	*MIR922*	*IER3*	*ETV1*	*SNX9*
ADCY3	*RHBDD1*	*RRP7A*	*PDE6B*	*HCG27*	*AGR3*	*ESYT2*
RAB10	*CCL20*	*RNU12*	*FAM53A*	*PIM1*	*MACC1*	*DYNC2I1*
MAPRE3	*NCL*	*BIK*	*SLBP*	*CNPY3*	*ITGB8*	*LOC389831*
AGBL5	*TEX44*	*MCAT*	*ABLIM2*	*CRIP3*	*MALSU1*	*MIR596*
TCF23	*PTMA*	*LOC730668*	*CPZ*	*VEGFA*	*OSBPL3*	*KBTBD11*
MRPL33	*GBX2*	*MIRLET7BHG*	*USP17L11*	*LINC01512*	*HOXA3*	*ZDHHC14*
MIR4263	*HDAC4*	*TBC1D22A*	*USP17L20*	*HSP90AB1*	*HIBADH*	*ARHGEF5*
FOSL2	*RNPEPL1*	*PLXNB2*	*CPEB2-DT*	*AARS2*	*TAX1BP1*	*EZH2*
WDR43	*LINC01881*	*SCO2*	*SLC34A2*	*RUNX2*	*CHN2*	*LOC155060*
FAM98A	*RBCK1*	*ARSA*	*TBC1D1*	*TNFRSF21*	*PRR15*	*REPIN1*

ATL2	*SLC52A3*	*SETD5*	*KLF3*	*PAQR8*	*ZNRF2*	*ZNF775*
SOS1	*CDC25B*	*CRELD1*	*CWH43*	*TRAM2*	*FKBP9*	*MUC12*
PPM1B	*PLCB1*	*IRAK2*	*DCUN1D4*	*KLHL31*	*NPSR1-AS1*	*SERPINE1*
EPCAM	*SPTLC3*	*VGLL4*	*CENPC*	*FAM83B*	*UBE2D4*	*ZNHIT1*
MSH2	*DSTN*	*TSEN2*	*UGT2B15*	*GUSBP4*	*LINC00957*	*MYL10*
MSH6	*OVOL2*	*ZNF860*	*CXCL5*	*KHDRBS2*	*WDR27*	*CUX1*
ACYP2	*LINC00261*	*UBP1*	*MTHFD2L*	*PTP4A1*	*LINC00242*	*SLC1A1*
SPTBN1	*MIR663AHG*	*PDCD6IP*	*ANXA3*	*SH3BGRL2*	*LOC154449*	*RASA4*
RTN4	*FRG1BP*	*VILL*	*ENOPH1*	*TPBG*	*FAM120B*	*LHFPL3-AS2*
EHBP1	*MLLT10P1*	*OXSR1*	*THAP9-AS1*	*PM20D2*	*FAM20C*	*KMT2E-AS1*
LINC00309	*DEFB115*	*CTNNB1*	*GPRIN3*	*PRDM1*	*ADAP1*	*SYPL1*
SLC1A4	*HM13-AS1*	*POMGNT2*	*STPG2*	*SLC2A12*	*UNCX*	*IFRD1*
SPRED2	*ID1*	*SLC6A20*	*UBE2D3*	*MYB*	*MICALL2*	*ZNF252P*
SLC25A51	*FAM27C*	*PTGER4P2-CDK2AP2P2*	*LOC286297*	*AQP7P1*	*MAFK*	*SPIN4*
NTRK2	*SPIN1*	*WNK2*	*PHF2*	*MIRLET7A1*	*TMEM184A*	*CXCR3*
SLC2A8	*NIBAN2*	*NAIF1*	*PTGES2*	*SPTAN1*	*SNX8*	*FGF16*
TUBB4B	*NRARP*	*NOXA1*	*ARRDC1*	*FAM157B*	*MIR4648*	*ATRX*
PLS3	*SLC39A4*					
