# Supplementary material for: Systemic genome-epigenome analysis captures a lineage-specific super-enhancer for MYB in gastrointestinal adenocarcinoma
Source: Mol Syst Biol. 2025 Apr 15;21(6):696–719. doi: 10.1038/s44320-025-00098-1 (PMC12130324; doi:10.1038/s44320-025-00098-1)
Supplement: Supplementary file 5 — Table EV4 [file 44320_2025_98_MOESM5_ESM.pdf]

Table EV4: 407 genes that are potential direct targets of MYB

ZNF185	*NDFIP2*	*RAB5A*	*UGCG*	*ACSL6*	*RASAL1*	*ENO1*	*PPP1R14C*
NOTCH3	*TGFBR3*	*MINDY2*	*PRDM1*	*DYNC2I2*	*PLEKHG6*	*CYB5B*	*RET*
SCN5A	*MUC17*	*NPTX2*	*MYEF2*	*PGD*	*HIPK2*	*MSLN*	*HERPUD1*
SLC22A3	*SLC25A36*	*AFDN*	*MPRIP*	*FLNB*	*ABCA1*	*ACVR1B*	*STEAP3*
TFAP2C	*DISC1*	*SECISBP2L*	*CEP55*	*GGCX*	*NRN1*	*NOS3*	*HASPIN*
ATP2B4	*SLC52A3*	*PRSS23*	*MELTF*	*NEURL1B*	*FAM81A*	*TRPV3*	*RIMBP2*
TENM2	*GCOM1*	*KLHL29*	*ADM*	*IGF1R*	*TRABD2A*	*BUB1*	*RIT1*
GSN	*AMOTL2*	*WWC2*	*CDKN3*	*KCTD15*	*GALNT3*	*NBEAL1*	*VEGFA*
INSIG1	*MYL9*	*HNRNPM*	*IL27RA*	*TMEM62*	*IDI1*	*MCM3*	*PIGC*
CMBL	*TNC*	*MCM5*	*TNFSF15*	*HGD*	*DAPK2*	*AP1S2*	*ZSWIM6*
DPYSL2	*HSD17B10*	*NEK2*	*ELAVL2*	*ZFAND5*	*DDX39A*	*B4GALNT2*	*HMBS*
CADPS	*ALPG*	*TMPRSS3*	*DRD2*	*CAPS2*	*ERN1*	*FYCO1*	*ATP6V0D1*
NTN4	*SLC35F5*	*MYO15B*	*APOL2*	*ARHGEF10L*	*CHAC1*	*PITX1*	*UACA*
BAHCC1	*FOSL2*	*SUSD6*	*TMSB10*	*PARK7*	*MSMO1*	*RNF144A*	*GLUL*
SLC6A19	*PLXND1*	*TANGO6*	*FBLIM1*	*MEF2D*	*TPD52L1*	*CDC20*	*FHOD3*
BMF	*HES6*	*RAB11FIP2*	*CAMTA2*	*RMND5A*	*ENPP1*	*CCND2*	*BUD13*
NFIC	*PTK7*	*MCC*	*NABP2*	*FRMD5*	*PLEKHG1*	*MARK1*	*LSM3*
MERTK	*LAMC2*	*JMY*	*ITGB6*	*SERTAD2*	*SLFN5*	*WSB1*	*MAPRE3*
TESC	*PPP1R9A*	*MXI1*	*PLB1*	*KCNK1*	*TSPAN2*	*KRT80*	*HECA*
TUBB4B	*GOLGA7B*	*IRF1*	*C15orf62*	*SLC22A23*	*CALCOCO1*	*CCDC9B*	*ALG10B*
SLC1A1	*TPCN1*	*GABRE*	*UBE2E2*	*ABCC2*	*ARRDC4*	*ATP5F1A*	*DSCC1*
CDCA7	*NFE2L1*	*MUC4*	*SLC25A33*	*PVRIG*	*FEN1*	*ALPP*	*ST6GAL2*
MUC6	*PBK*	*ARHGAP6*	*HSBP1*	*SEC22B*	*DNTTIP1*	*ZNF12*	*AHCY*
IFI6	*DUSP4*	*GOLGA3*	*RAB30*	*XRCC5*	*CDKN2B*	*MUC12*	*USP13*
GPC4	*DLX2*	*LATS2*	*CD44*	*MGST2*	*CD320*	*LSM4*	*PAX8*
LPIN2	*TUBA1C*	*FGFR1*	*B4GALNT3*	*TRIP13*	*DLX3*	*REN*	*TNFRSF21*
SPEG	*KDM6B*	*SLC15A1*	*HBP1*	*SMARCE1*	*WFDC3*	*RASGEF1B*	*SH3RF1*
CCL24	*TVP23B*	*JAG1*	*ZNF217*	*CXCL16*	*KIT*	*CDK18*	*PUF60*
HID1	*LAMA5*	*PTPRF*	*VPS35L*	*CLDN9*	*L1TD1*	*TMEM63A*	*KEAP1*
FGL2	*SLC9A3*	*CNGA1*	*WDR44*	*UTP14A*	*CPXM2*	*MAD2L1*	*TNFAIP1*
PCSK5	*PKP1*	*GDF10*	*KRT15*	*MYB*	*NABP1*	*LRP11*	*MRPL11*
NRP1	*FAM171A1*	*SOX8*	*KIF22*	*FNDC3A*	*PPFIBP1*	*GINS2*	*MRPL4*
CCNB1	*ATP6V1C2*	*ARHGAP18*	*PC*	*NT5C2*	*EPHB3*	*KPNA2*	*GNAQ*
ARHGAP23	*STEAP2*	*GOT2*	*MEF2C*	*ENPP5*	*ST3GAL4*	*DEPDC1B*	*DEPDC7*
UNC13D	*PPL*	*SH3PXD2B*	*KLF10*	*MFSD12*	*MAP4K4*	*TMEM106B*	*TOR3A*
ACAA2	*PKM*	*PNPLA3*	*FAM102A*	*SAE1*	*TOM1L2*	*ATP1B1*	*NCOR2*
FKBP14	*NR6A1*	*PHYH*	*HMGCR*	*GRIK4*	*CGN*	*CHI3L1*	*PRSS8*
F13A1	*NECTIN4*	*COQ2*	*UBASH3B*	*TNS4*	*PKHD1*	*CLMN*	*PREX1*
MYLIP	*DPY19L1*	*CEACAM6*	*PTTG1*	*FYN*	*STYXL2*	*KLF4*	*PPP1R26*
GCNT1	*NTSR1*	*SORL1*	*RNASET2*	*CHRM3*	*CORO2B*	*UHRF1*	*NOTUM*
SIT1	*FAM83D*	*F8*	*SLC2A1*	*LAT2*	*PRDX2*	*ACSF2*	*CFAP53*
SERP2	*CPVL*	*CBFA2T2*	*P2RX4*	*BICDL1*	*FGD3*	*TUBB6*	*BNIP2*
HAS2	*SHF*	*RRM1*	*TRIM29*	*MAP7D2*	*LRP3*	*PLEKHG5*	*CAB39L*
VAC14	*ZKSCAN1*	*ABTB2*	*UBE2S*	*CCDC146*	*CASK*	*PSMA6*	*KSR1*
CASP7	*ZFP36L2*	*CYC1*	*GSDMB*	*SAMD3*	*TINAGL1*	*RALGPS1*	*HMGB1*
FCGBP	*OTULINL*	*HMOX2*	*CNNM4*	*MYRIP*	*TMPRSS2*	*FASN*	*SMOC2*
VGLL4	*FTH1*	*PCBP3*	*ZNF608*	*PTDSS1*	*TRIM28*	*KIF20A*	*BAMBI*
RACGAP1	*ENPP4*	*GPR107*	*PRKX*	*FAM131B*	*PELI1*	*OGN*	*PLA2R1*

ISG20	*PLD1*	*PLA2G15*	*PGS1*	*PRSS22*	*CENPE*	*ACAP2*	*FAM111B*
NUF2	*PPP1CA*	*ATP10B*	*RNF103*	*FGFR4*	*LDLR*	*VSIR*	*WDR26*
SAMHD1	*OLFM1*	*LAMB2*	*PRR15L*	*MMAB*	*CLIP4*	*MGAT3*	
