# Supplementary material for: Systemic genome-epigenome analysis captures a lineage-specific super-enhancer for MYB in gastrointestinal adenocarcinoma
Source: Mol Syst Biol. 2025 Apr 15;21(6):696–719. doi: 10.1038/s44320-025-00098-1 (PMC12130324; doi:10.1038/s44320-025-00098-1)
Supplement: Supplementary file 6 — Table EV5 [file 44320_2025_98_MOESM6_ESM.pdf]

Table EV5: Primers used in this study

Primers used to clone enhancer fragments into the luciferase reporter construct pGL3-promoter		Notes	
MYB_e1_MluI_F	ATAT ACGCGT TACTGTTTTAAGCTCAGTAC	for single e1	
MYB_e1_XhoI_R	ATAT CTCGAG CATCCTGCGTTCTCATTTTG		
MYB_e2_MluI_F	ATAT ACGCGT CTTGGCACAGAGGGGAGTCT	for single e2	
MYB_e2_XhoI_R	ATAT CTCGAG GATAGGTTTATCCCCTTCAC		
MYB_e3_MluI_F	ATAT ACGCGT GAAGTTTTCAGAAAAGGAGG	for single e3	
MYB_e3_XhoI_R	ATAT CTCGAG TCAAGCCCATGGCCTTGCCT		
MYB_e4_MluI_F	ATAT ACGCGT TCAGCCCATAACAGTCACCA	for single e4	
MYB_e4_XhoI_R	ATAT CTCGAG TGTTGCAGGTGGCATTAGTT		
MYB_e5_MluI_F	ATAT ACGCGT GGGAGACACCACGCACATTC	for single e5	also as the e4+e5+e6+e7_F
MYB_e5_XhoI_R	ATAT CTCGAG AAGGAAGCCCCTCCCTCCAC		
MYB_e6_MluI_F	ATAT ACGCGT GTCAGGAAATAATTAGCAAA	for single e6	
MYB_e6_XhoI_R	ATAT CTCGAG GCAGGTAGTGAGGAATTCCT		
MYB_e7_MluI_F	ATAT ACGCGT GGAAACAGACGCAGAAACCT	for single e7	
MYB_e7_XhoI_R	ATAT CTCGAG AGAGGAAATCTATTTCCACT		also as the e4+e5+e6+e7_R
MYB_e4_KpnI_F	ATAT GGTACC TCAGCCCATAACAGTCACCA	for duplicate e4	
MYB_e4_MluI_R	ATAT ACGCGT TGTTGCAGGTGGCATTAGTT		
MYB_e3_KpnI_F	ATAT GGTACC GAAGTTTTCAGAAAAGGAGG	for cloning e3 into e4 pGL3-promoter plasimd	
MYB_e3_MluI_R	ATAT ACGCGT TCAAGCCCATGGCCTTGCCT		

**Primers for site-directed deletion of motif sequence**		Notes	Tm
EBF_deletion_F	GGGAGGTGAAGCGGAAGT	for 1st EBF motif deletion of e4	64°C
EBF_deletion_R	ATCAAAGTTTTGGGGCTTTTTTTTTTC		
ETS_deletion_F	TGATCTTCACCATTAAATTTTTTTATAGATAGCAAAGTC	for ETS motif deletion of e4	67°C
ETS_deletion_R	GCTTCACCTCCCCAGGGA		
HNF4_deletion_F	CATGCTTCCACCTGCATC	for HNF4A motif deletion of e4	61°C
HNF4_deletion_R	GCTATCTATAAAAAAATTTAATGGTGAAG		
ASCL_deletion_F	GCAGAACAAAGCACCAGAG	for ASCL_famlies motif deletion of e4	64°C
ASCL_deletion_R	CCTCTGTGCTTTGGATGC		
2st SOX_deletion_F	CACCAGAGAAAAATAGCTGACCAG	for 2st SOX_famlies motif deletion of e4	66°C
2st SOX_deletion_R	TCTGCCAGCTGCCTCTGT		
SNAI_deletion_F	CAGCTTGTTTTTCATCTTG	for SNAI_famlies motif deletion of e4	57°C
SNAI_deletion_R	TACACAGGCAATTTAAGAG		

**sgRNAs for CRISPR-mediated enhancer repression**		Notes
**Targeting to *MYB-* SE enhancer cluster**		
sg_NC1_F	CACCGATCGTTTCCGCTTAACGGCG	Negative control; also used for CRISPRa
sg_NC1_R	AAACCGCCGTTAAGCGGAAACGATC	
sg_NC2_F	CACCGCTGAGTGAAAAATAAAAGTT	Negative control; also used for CRISPRa
sg_NC2_R	AAACAACTTTTATTTTTCACTCAGC	
sg_e1.1_F	CACCGACAGGGCTGGCTCTCTGGCT	
sg_e1.1_R	AAACAGCCAGAGAGCCAGCCCTGTC	
sg_e1.2_F	CACCGATATCTCCACTCTGTCTATT	
sg_e1.2_R	AAACAATAGACAGAGTGGAGATATC	
sg_e2.1_F	CACCGAGGGTGTGGCTAGAGAGTGG	
sg_e2.1_R	AAACCCACTCTCTAGCCACACCCTC	
sg_e2.2_F	CACCGACATCCTTCCTTAGGGCTG	
sg_e2.2_R	AAACCAGCCCTAAGGAAGGATGTC	
sg_e3.1_F	CACCGCAGTACTTGACAGAGGGTG	
sg_e3.1_R	AAACCACCCTCTGTCAAGTACTGC	
sg_e3.2_F	CACCGAAAGAGAAAGTTTTAAGAGT	
sg_e3.2_R	AAACACTCTTAAAACTTTCTCTTTC	
sg_e4.1_F	CACCGGCAACGACGTGGATAGGAC	
sg_e4.1_R	AAACGTCCTATCCACGTCGTTGCC	
sg_e4.2_F	CACCGTTTGGATGCAGGTGGAAGCA	
sg_e4.2_R	AAACTGCTTCCACCTGCATCCAAAC	
sg_e4.3_F	CACCGCTCTTAAATTGCCTGTGTAC	also called as sg_e4#3_F
sg_e4.3_R	AAACGTACACAGGCAATTTAAGAGC	also called as sg_e4#3_R
sg_e4.4_F	CACCGCTCTGTGCTTTGGATGCAGG	also called as sg_e4#4_F
sg_e4.4_R	AAACCCTGCATCCAAAGCACAGAGC	also called as sg_e4#4_R
sg_e5.1_F	CACCGGCTCCAGGCCAGGGGCTCT	
sg_e5.1_R	AAACAGAGCCCCTGGCCTGGAGCC	
sg_e5.2_F	CACCGTTGTGCTTTTGTCTTGGACT	
sg_e5.2_R	AAACAGTCCAAGACAAAAGCACAAC	
sg_e6.1_F	CACCGATTGCTCCTGGTTACCTTG	
sg_e6.1_R	AAACCAAGGTAACCAGGAGCAATC	
sg_e6.2_F	CACCGAAAACTGGAACATATTGTGG	
sg_e6.2_R	AAACCCACAATATGTTCCAGTTTTC	
sg_e7.1_F	CACCGCCTGAGAGAGCTTGAGCAAT	
sg_e7.1_R	AAACATTGCTCAAGCTCTCTCAGGC	
sg_e7.2_F	CACCGCTGCCAGGCAGTGCAGAGCT	
sg_e7.2_R	AAACAGCTCTGCACTGCCTGGCAGC	

**Targeting to *ASCL2* candidate enhancers**		Notes
sg_e1_F	CACCGCACGTGCCCAAGCTCCCGT	
sg_e1_R	AAACACGGGAGCTTGGGCACGTGC	
sg_e2_F	CACCGCCTCCAGTGGACCCAGGGG	
sg_e2_R	AAACCCCCTGGGTCCACTGGAGGC	
sg_e3_F	CACCGTTGTTCAGCGCAGTCCGCAG	
sg_e3_R	AAACCTGCGGACTGCGCTGAACAAC	

**Targeting to *GATA6* candidate enhancers**		Notes
sg_e1_F	CACCGTCTGCAGTAAAGCAGTGCAA	
sg_e1_R	AAACTTGCACTGCTTTACTGCAGAC	
sg_e2_F	CACCGTAAAGATCAGAGGAAGAGG	
sg_e2_R	AAACCCTCTTCCTCTGATCTTTAC	

**Targeting to *ZFP36L2* candidate enhancers**		Notes
sg_e1_F	CACCGCAGATGGAGACATTACGCCG	
sg_e1_R	AAACCGGCGTAATGTCTCCATCTGC	
sg_e2_F	CACCGTGTGACTGTAAAGTAATCCA	
sg_e2_R	AAACTGGATTACTTTACAGTCACAC	

sgRNAs for CRISPR-mediated enhancer activation		Notes
sg_e4.5_F	CACCGTTTGGATGCAGGTGGAAGCA	also called as sg_e4#5_F
sg_e4.5_R	AAACTGCTTCCACCTGCATCCAAAC	also called as sg_e4#5_R
sg_e4.6_F	CACCGTAGAGCAACGCCTAGACAA	also called as sg_e4#6_F
sg_e4.6_R	AAACTTGTCTAGGCGTTGCTCTAC	also called as sg_e4#6_R

sgRNAs for CRISPR-mediated motif cutting within enhancer e4		Notes
sg_e4_HNF4_F	CACCGATTGACAATGTTTTACCCAG	HNF4 motif
sg_e4_HNF4_R	AAACCTGGGTAAAACATTGTCAATC	
sg_e4_EBF_F	CACCGCTCTTAAATTGCCTGTGTAC	EBF motif
sg_e4_EBF_R	AAACGTACACAGGCAATTTAAGAGC	
sg_e4_SNAI_F	CACCGCAAAACTTTGATCCCTGGGG	SNAI motif
sg_e4_SNAI_R	AAACCCCCAGGGATCAAAGTTTTGC	
sg_e4_ASCL_F	CACCGATCCAAAGCACAGAGGCAGC	ASCL motif
sg_e4_ASCL_R	AAACGCTGCCTCTGTGCTTTGGATC	

RT-qPCR primers		Target genes
RT_HPRT1_F	GACCAGTCAACAGGGGACAT	*HPRT1*
RT_HPRT1_R	CCTGACCAAGGAAAGCAAAG	
RT_R28S_F	CGATCCATCATCCGCAATG	*R28S*
RT_R28S_R	AGCCAAGCTCAGCGCAAC	
RT_MYB_F	GCACCAGCATCAGAAGATGA	*MYB*
RT_MYB_R	CTTTCCACAGGATGCAGGTT	
RT_MYB_3'UTR_F	TTCATGAATGGGAGAAGAACCT	*MYB_* 3'UTR
RT_MYB_3'UTR_R	TTGGGTGAAATCCAAGAAGAC	
RT_HNF4A_F	AGAACCACATGTACTCCTGCA	*HNF4A*
RT_HNF4A_R	CTTCCTTCTTCATGCCAGCC	
RT_EBF1_F	GGGATGATGGGCGTGAATTC	*EBF1*
RT_EBF1_R	GTGGTGATACGCTGCTTGAG	
RT_HNF4G_F	CGGTGTCAACTGTCTGTGTG	*HNF4G*
RT_HNF4G_R	CAACACATTGCCGACTGAAC	
RT_GATA6_F	TTCCCATGACTCCAACTTCC	*GATA6*
RT_GATA6_R	CGCCTATGTAGAGCCCATCT	
ZFP36L2_F_RT	CTTCTGTCCGCCTTCTACGA	*ZFP36L2*
ZFP36L2_R_RT	GCCTTCTTGTCCAGCATGTT	
BCLAF1_F_RT	AGGGGATGGGATTGTTGAAGA	*BCLAF1*
BCLAF1_R_RT	AATGGTGGGTGCAAGTTCTG	
MAP7_F_RT	TGCCTCAGGTAGATGGTGTT	*MAP7*
MAP7_R_RT	GAAAACGGGTGGAGGGGAT	
IL20RA_F_RT	GGGGTTATATGTGCAGATGGA	*IL20RA*
IL20RA_R_RT	CTGGGATCAAAGGGGTGACT	
HBS1L_F_RT	GAGTGCAGAGTTTGAAGGACA	*HBS1L*
HBS1L_R_RT	TGCTGACTGAGGATAAGAACTTT	
ALDH8A1_F_RT	CATCTGCGTGCACTCTGAC	*ALDH8A1*
ALDH8A1_R_RT	CCCAGGCCGAGTCTAAAGA	
AHI1_F_RT	ATGCAGGAGAACGAGGATGT	*AHI1*
AHI1_R_RT	TGGCCACACAATTCTCTCATG	
NOTCH3_RT_F	GCAAATGGAGGTCGTTGCA	*NOTCH3*
NOTCH3_RT_R	AGCCACCACTGAACTCTGG	
ST3GAL4_RT_F	TCCTGGTAGCTTTCAAGGCA	*ST3GAL4*
ST3GAL4_RT_R	AGATGAGGGGAGGCTGTTTC	
B4GALT1_RT_F	ATCATTCCATTCCGCAACCG	*B4GALT1*
B4GALT1_RT_R	ACATTGAGGAGCTTAGCACG	
MFNG_RT_F	CCTCTTTCACTCCCACCTGG	*MFNG*
MFNG_RT_R	GGGGCCCTGTAGCTTAATGA	
ASCL2_RT1_F	GTGAAGCTGGTGAACTTGGG	*ASCL2*
ASCL2_RT1_R	CCACCTTGCTCAGCTTCTTG	
ASCL2_RT2_F	TAACTTGAGCTGCTGGAGGG	*ASCL2* _3'UTR
ASCL2_RT2_R	CAGGGGTCCAGGTCATCTTT	

ChIP-qPCR primers	
ChIP_NCR_F	TGGGTGGTGTCATCTGGTAA
ChIP_NCR_R	GGATGGAATGGATCAGATGG
ChIP_NCR2_F	GCTGCTTTAGTGGCTGTTCC
ChIP_NCR2_R	GCAGCTGCCGTCTTAACTGT
MYB_e4_ChIP_F	AAAGTCCATGCTTCCACCTG
MYB_e4_ChIP_R	TGCCACCAAGATGAAAAACA
MYB_e4_ChIP_2F	TCTCCTGCTACTTGTGTGGG
MYB_e4_ChIP_2R	CTGGTAGAGCAACGCCTAGA

Primers for shRNA	
shNC_F	CCGG GCAAGCTGACCCTGAAGTTCA CTCGAG TGAACTTCAGGGTCAGCTTGC TTTTTG
shNC_R	AATTCAAAAA GCAAGCTGACCCTGAAGTTCA CTCGAG TGAACTTCAGGGTCAGCTTGC
shMYB-3F	CCGGGCTCCTAATGTCAACCGAGAACTCGAGTTCTCGGTTGACATTAGGAGCTTTTTG
shMYB-3R	AATTCAAAAAGCTCCTAATGTCAACCGAGAACTCGAGTTCTCGGTTGACATTAGGAGC
shMYB-4F	CCGGCCCTCTCATCTAGTAGAAGATCTCGAGATCTTCTACTAGATGAGAGGGTTTTTG
shMYB-4R	AATTCAAAAACCCTCTCATCTAGTAGAAGATCTCGAGATCTTCTACTAGATGAGAGGG
shMYB-5F	CCGGGCATCAGAAGATGAAGACAATCTCGAGATTGTCTTCATCTTCTGATGCTTTTTG
shMYB-5R	AATTCAAAAAGCATCAGAAGATGAAGACAATCTCGAGATTGTCTTCATCTTCTGATGC
shMYB-6F	CCGGCCAGATTGTAAATGCTCATTTCTCGAGAAATGAGCATTTACAATCTGGTTTTTG
shMYB-6R	AATTCAAAAACCAGATTGTAAATGCTCATTTCTCGAGAAATGAGCATTTACAATCTGG
shEBF1-1F	CCGG GCAGTCTCTGATAACATGTTT CTCGAG AAACATGTTATCAGAGACTGC TTTTTG
shEBF1-1R	AATTCAAAAA GCAGTCTCTGATAACATGTTT CTCGAG AAACATGTTATCAGAGACTGC
shEBF1-2F	CCGG GCTCTATACAAGGGACACTAT CTCGAG ATAGTGTCCCTTGTATAGAGC TTTTTG
shEBF1-2R	AATTCAAAAA GCTCTATACAAGGGACACTAT CTCGAGA TAGTGTCCCTTGTATAGAGC
shHNF4A-1F	CCGGCGAGCAGATCCAGTTCATCAACTCGAGTTGATGAACTGGATCTGCTCGTTTTTG
shHNF4A-1R	AATTCAAAAACGAGCAGATCCAGTTCATCAACTCGAGTTGATGAACTGGATCTGCTCG
shHNF4A-2F	CCGGCCATCACCAAGCAGGAAGTTACTCGAGTAACTTCCTGCTTGGTGATGGTTTTTG
shHNF4A-2R	AATTCAAAAACCATCACCAAGCAGGAAGTTACTCGAGTAACTTCCTGCTTGGTGATGG
HNF4G-shRNA-1F	CCGGGCACATTTGATGGCAGCAACACTCGAGTGTTGCTGCCATCAAATGTGCTTTTTG

HNF4G-shRNA-1R	AATTCAAAAAGCACATTTGATGGCAGCAACACTCGAGTGTTGCTGCCATCAAATGTGC
HNF4G-shRNA-2F	CCGGGGTGGAATGGGCTAAATATATCTCGAGATATATTTAGCCCATTCCACCTTTTTG
HNF4G-shRNA-2R	AATTCAAAAAGGTGGAATGGGCTAAATATATCTCGAGATATATTTAGCCCATTCCACC
HNF4G-shRNA-3F	CCGGGCAGAGCATCACGTGGCAAATCTCGAGATTTGCCACGTGATGCTCTGCTTTTTG
HNF4G-shRNA-3R	AATTCAAAAAGCAGAGCATCACGTGGCAAATCTCGAGATTTGCCACGTGATGCTCTGC
