# Supplementary figures and images for: Systemic genome-epigenome analysis captures a lineage-specific super-enhancer for MYB in gastrointestinal adenocarcinoma
Source: Mol Syst Biol. 2025 Apr 15;21(6):696–719. doi: 10.1038/s44320-025-00098-1 (PMC12130324; doi:10.1038/s44320-025-00098-1)

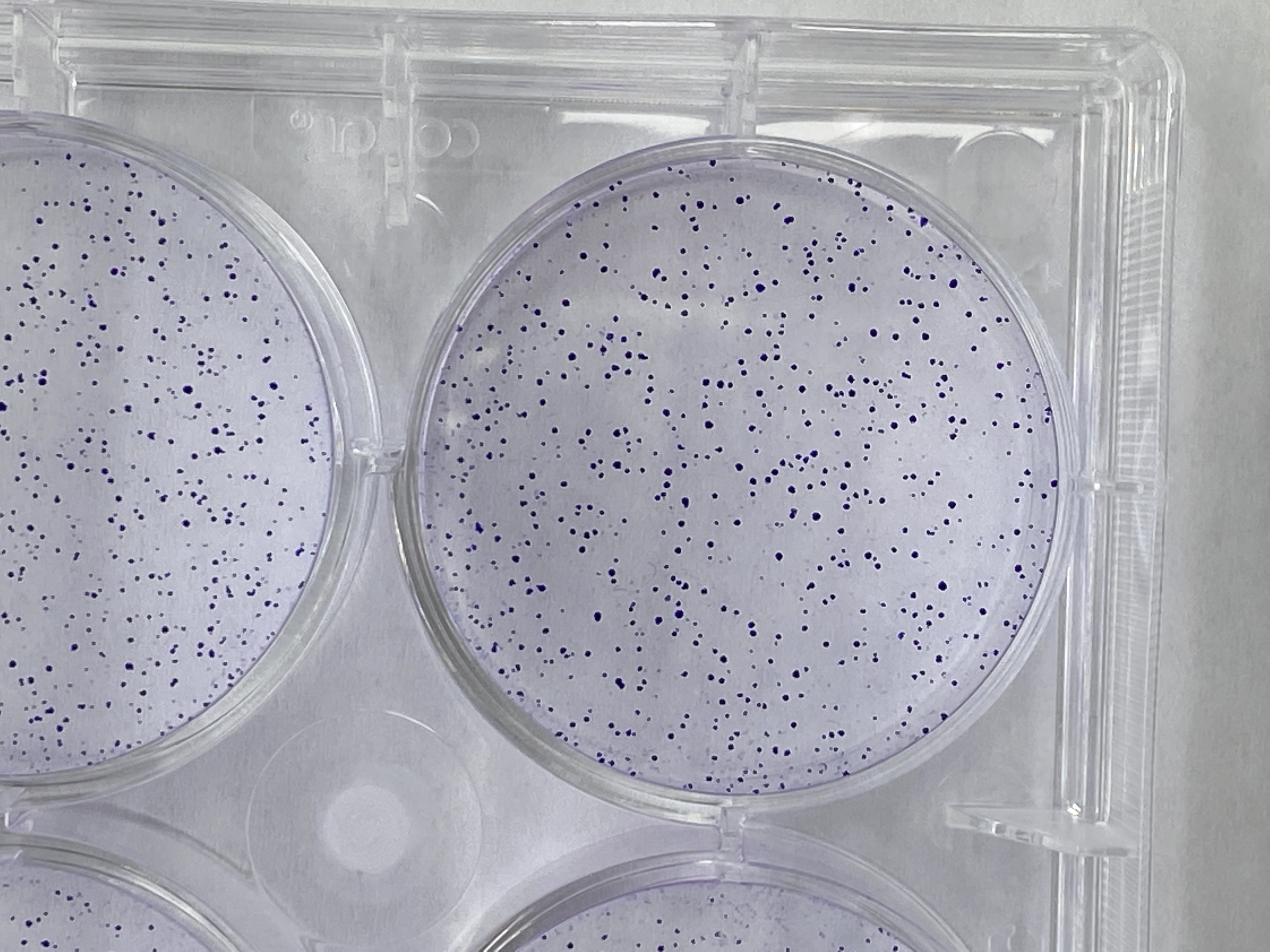

Supplement: Supplementary file 11 — Source data Fig. 6 [file 44320_2025_98_MOESM11_ESM.zip › Figure6/6C/CRISPRi#e4-3.jpg]

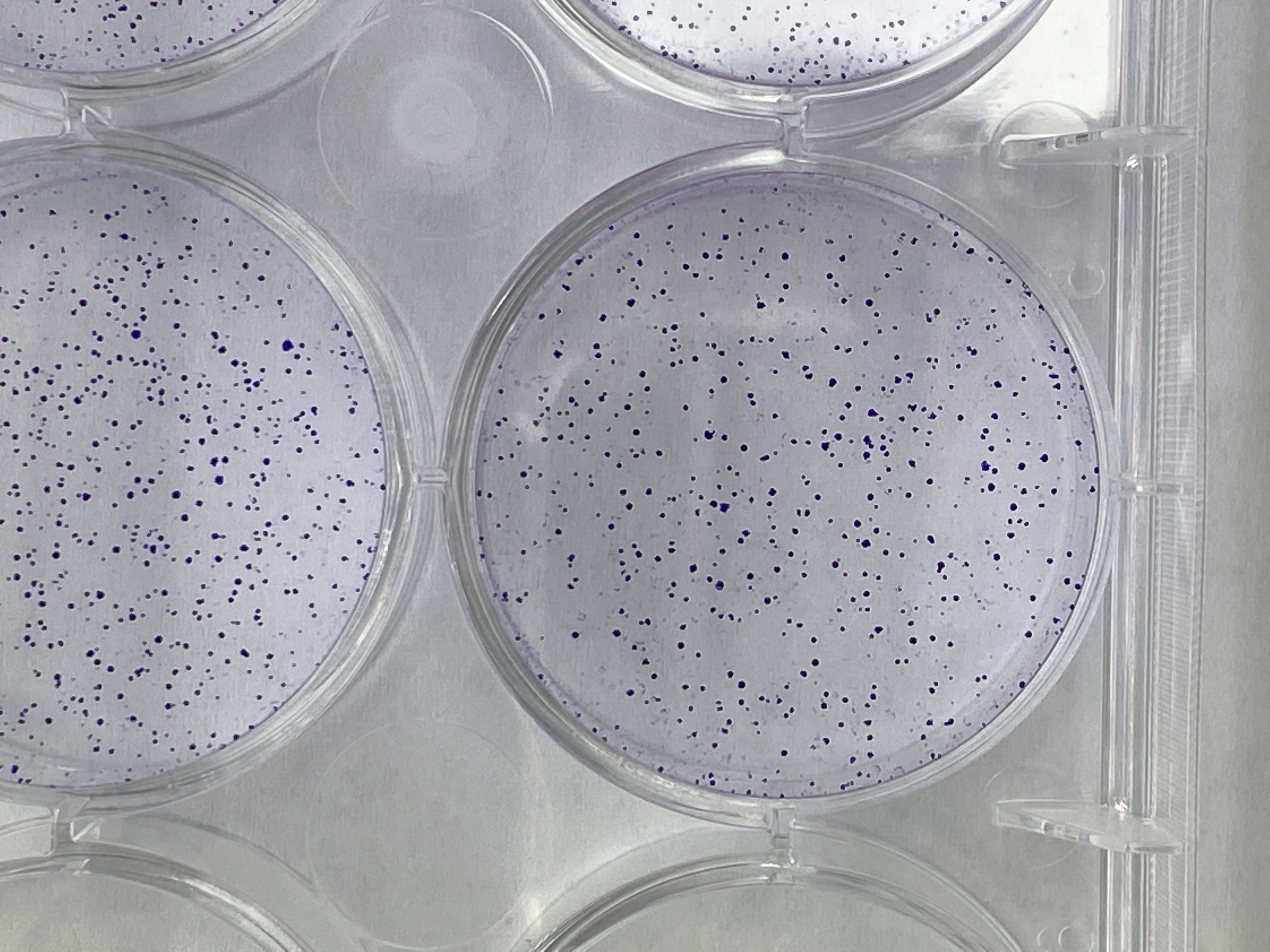

Supplement: Supplementary file 11 — Source data Fig. 6 [file 44320_2025_98_MOESM11_ESM.zip › Figure6/6C/CRISPRi#e4-4.jpg]

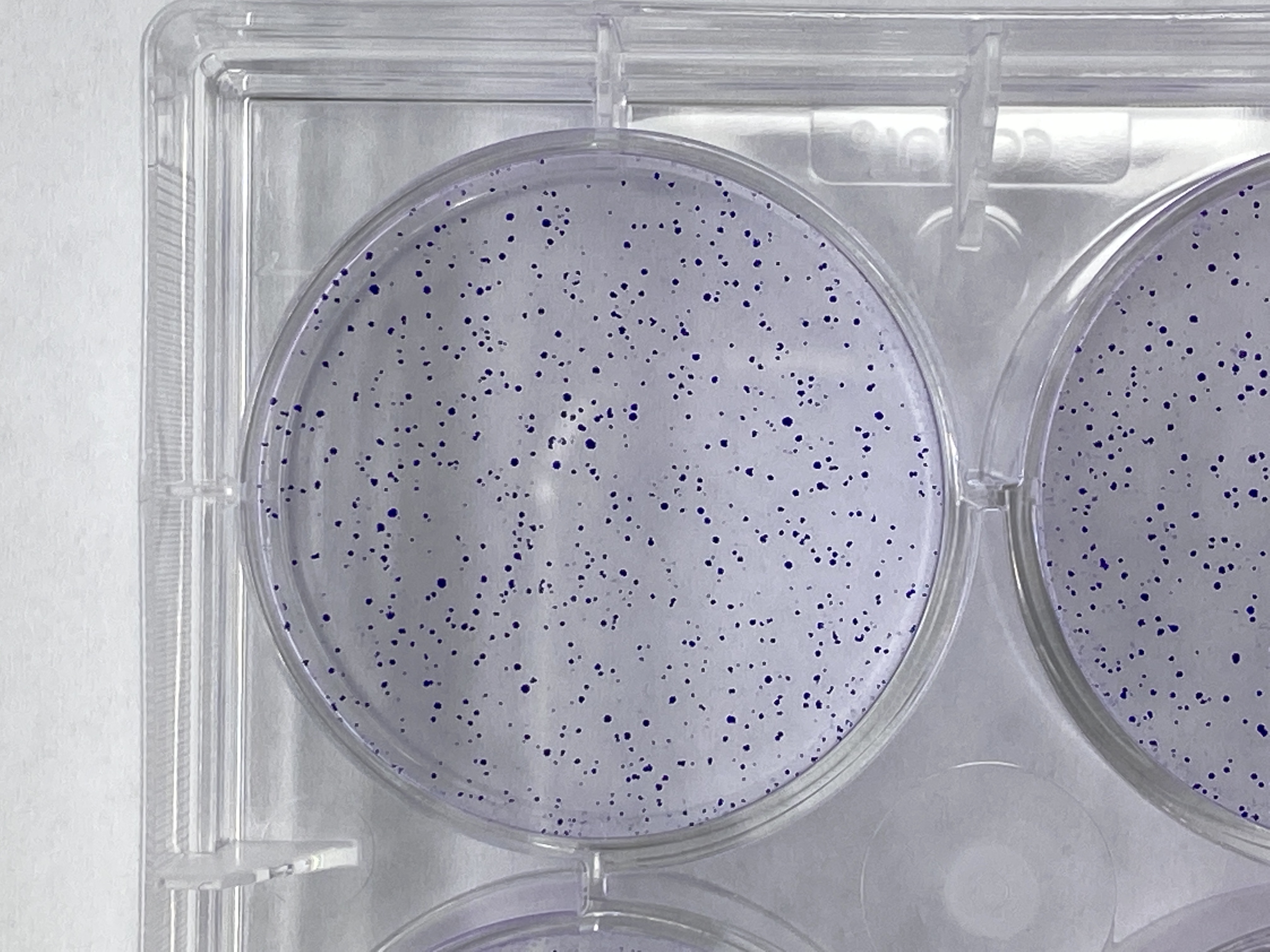

Supplement: Supplementary file 11 — Source data Fig. 6 [file 44320_2025_98_MOESM11_ESM.zip › Figure6/6C/CRISPRi#NC-1.jpg]

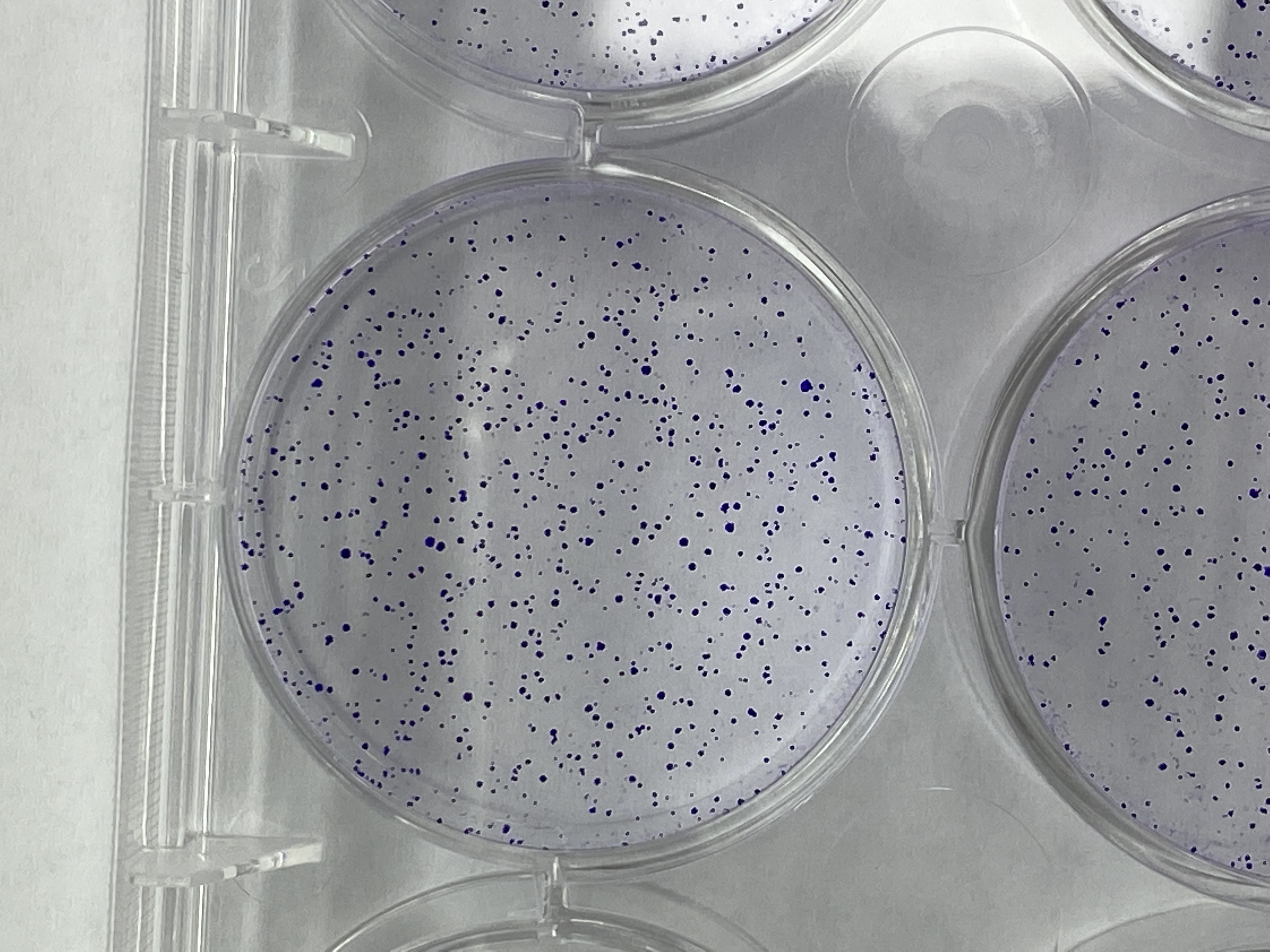

Supplement: Supplementary file 11 — Source data Fig. 6 [file 44320_2025_98_MOESM11_ESM.zip › Figure6/6C/CRISPRi#NC-2.jpg]

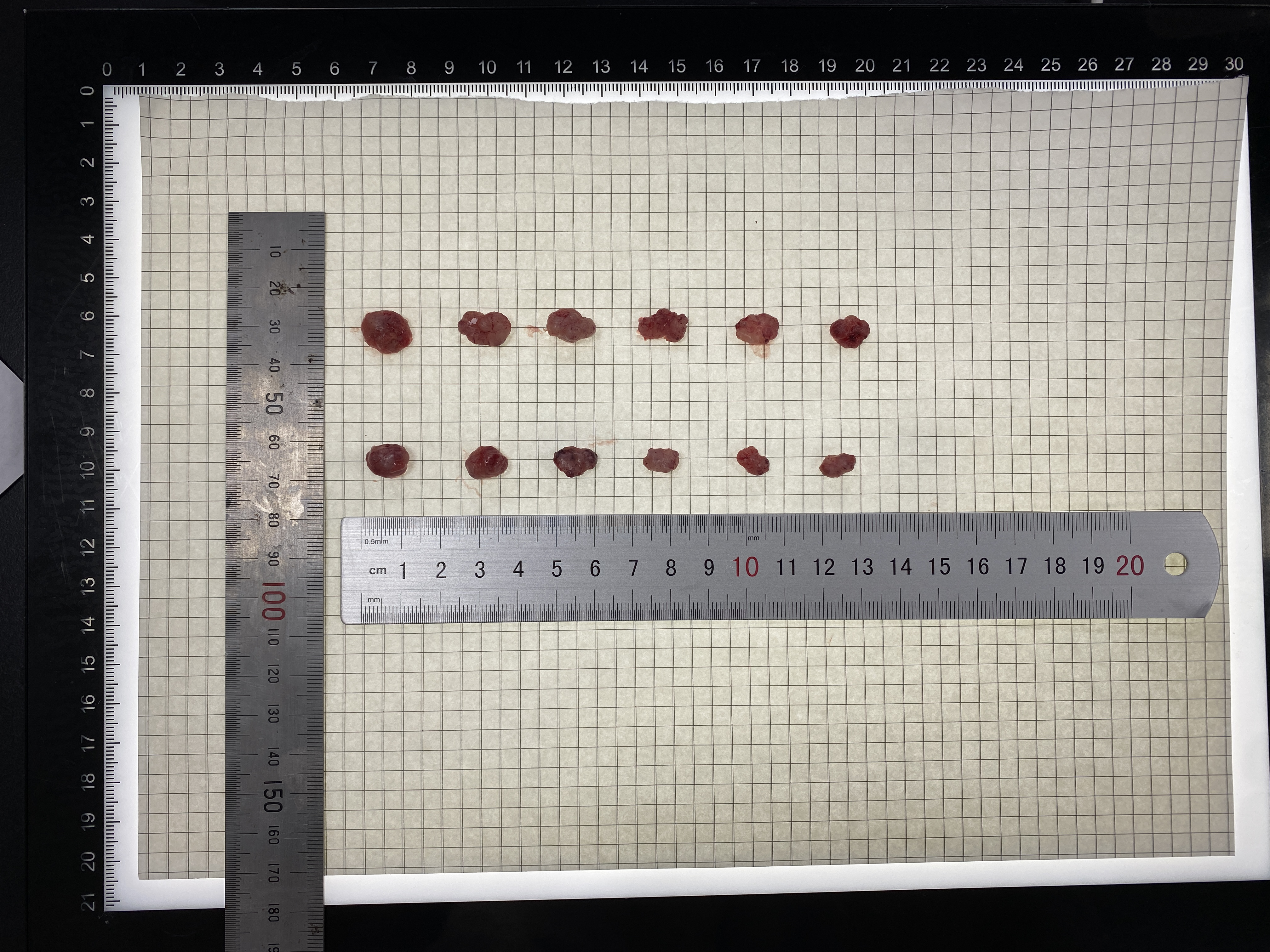

Supplement: Supplementary file 11 — Source data Fig. 6 [file 44320_2025_98_MOESM11_ESM.zip › Figure6/6F/6F_tumor.tif]

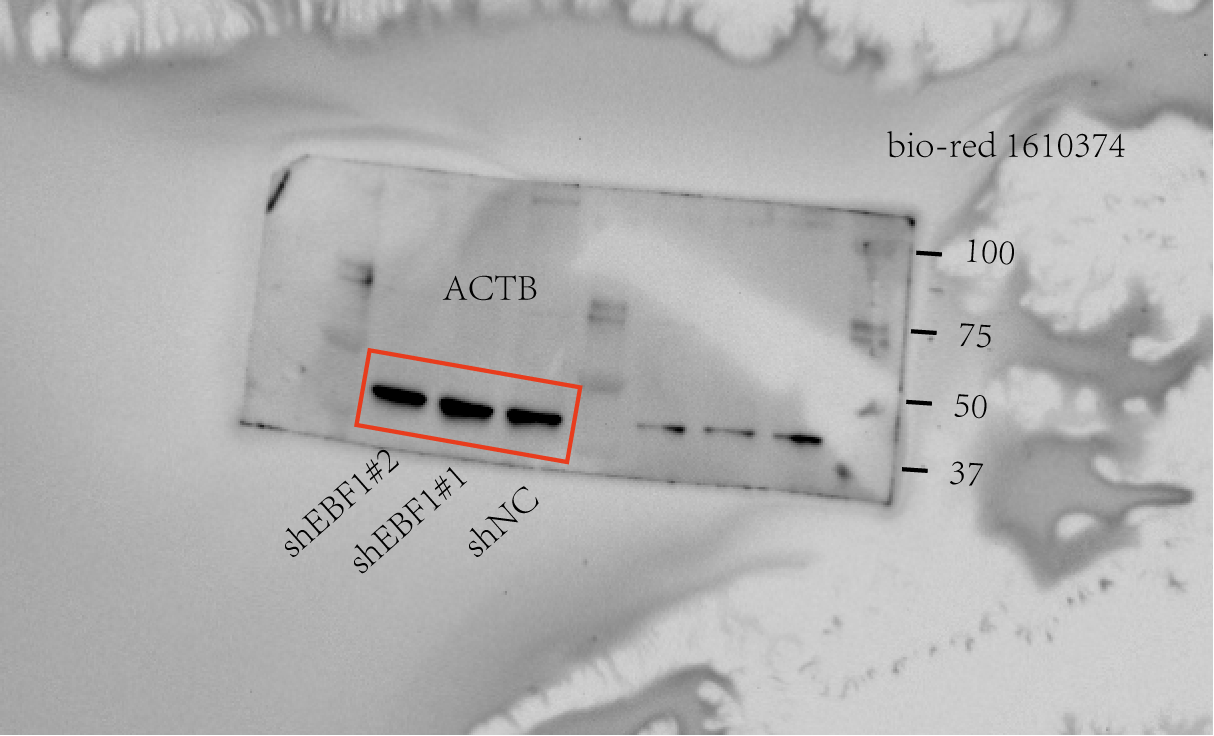

Supplement: Supplementary file 12 — EV and Appendix Figure Source Data [file 44320_2025_98_MOESM12_ESM.zip › SD for EV and Appendix figures/Figure EV4/EV4E/shEBF1-ACTB.tif]

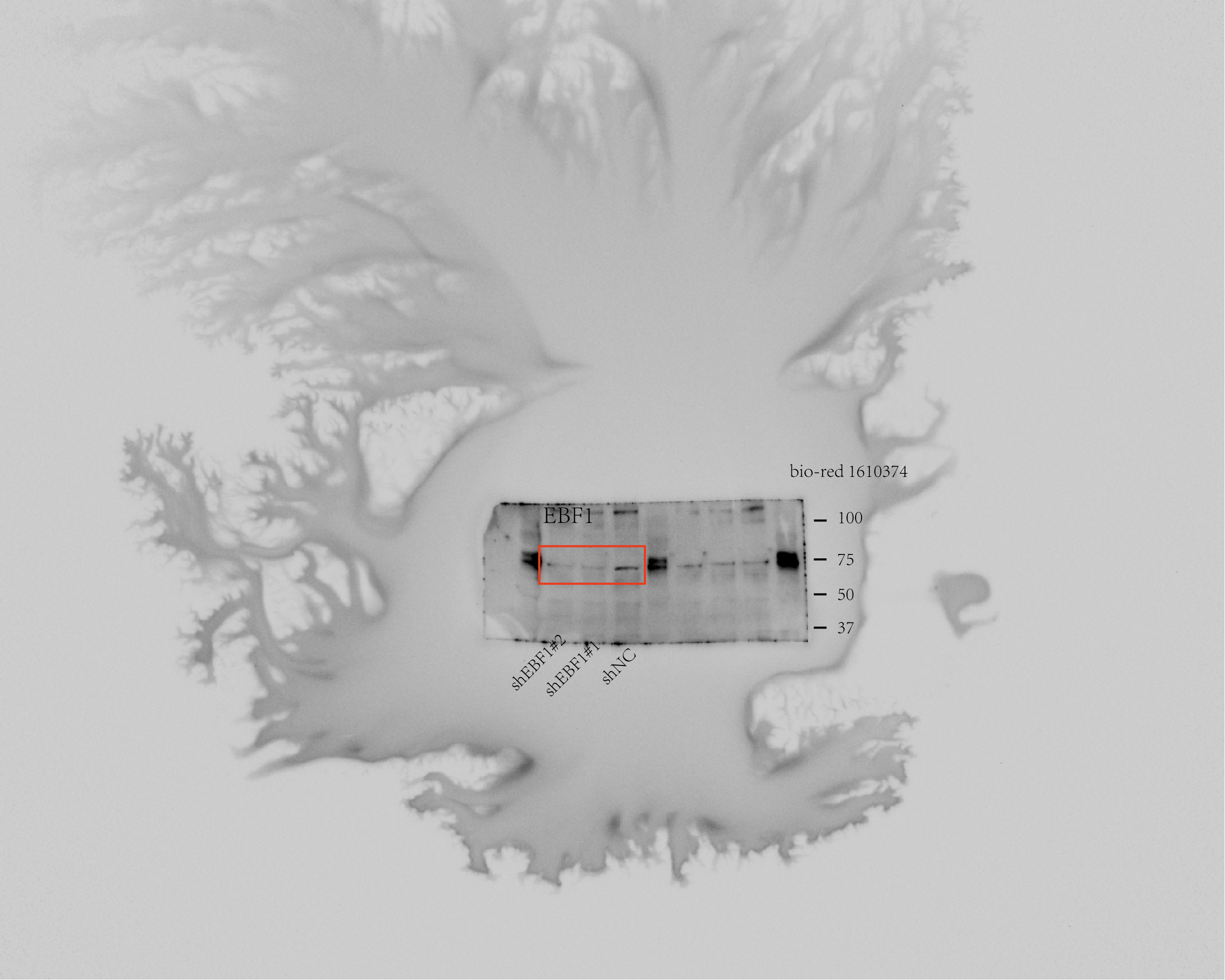

Supplement: Supplementary file 12 — EV and Appendix Figure Source Data [file 44320_2025_98_MOESM12_ESM.zip › SD for EV and Appendix figures/Figure EV4/EV4E/shEBF1-EBF1.tif]

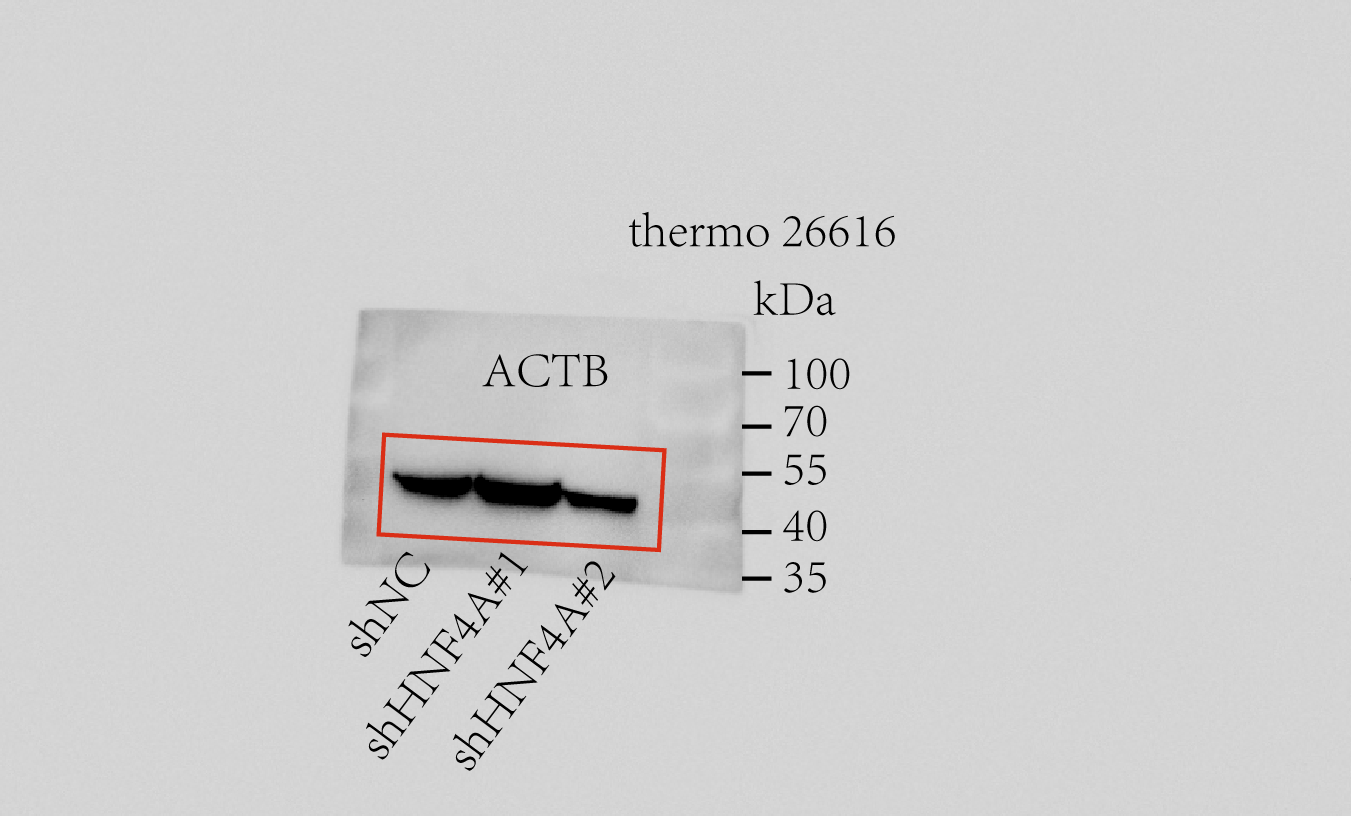

Supplement: Supplementary file 12 — EV and Appendix Figure Source Data [file 44320_2025_98_MOESM12_ESM.zip › SD for EV and Appendix figures/Figure EV4/EV4E/shHNF4A-ACTB.tif]

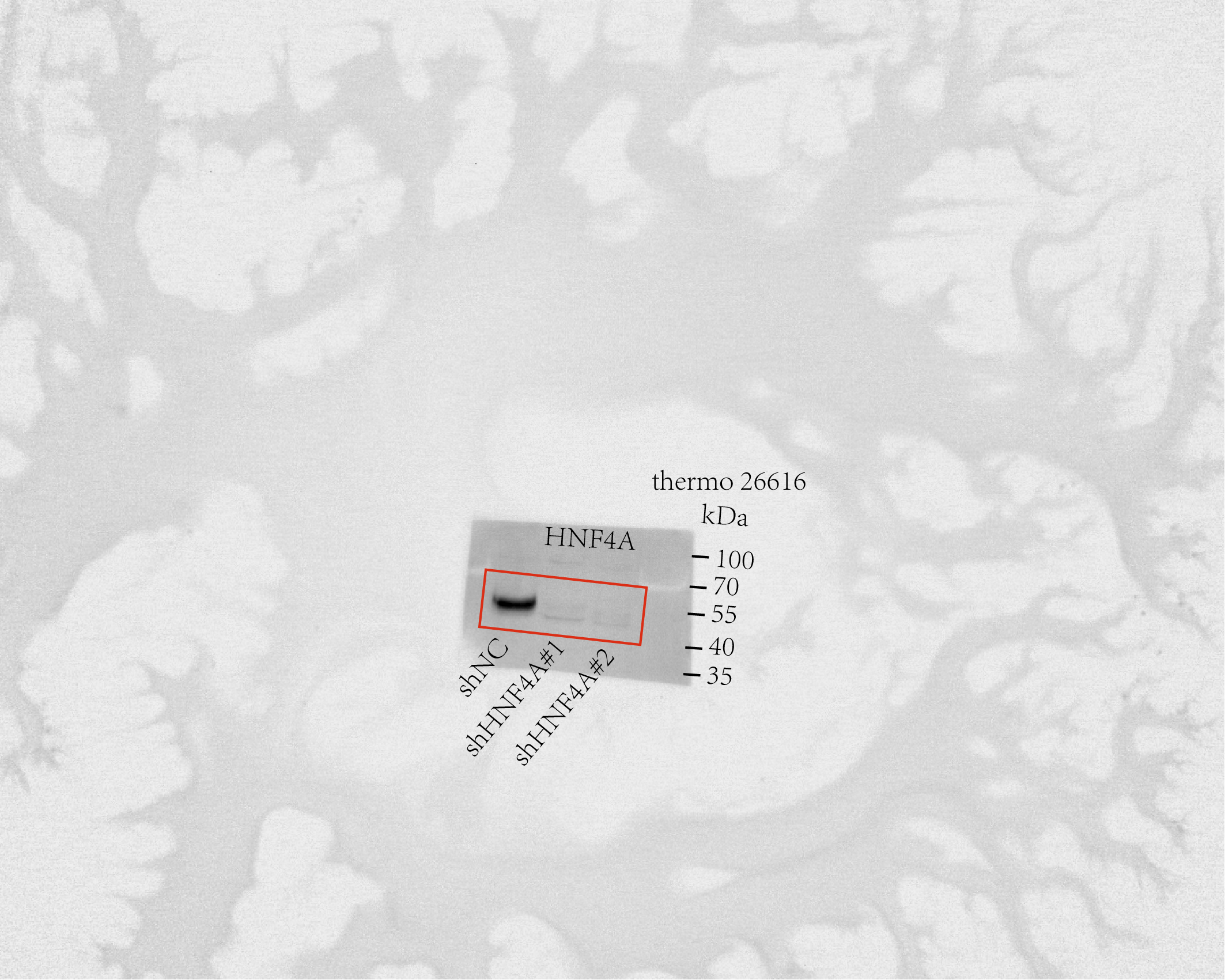

Supplement: Supplementary file 12 — EV and Appendix Figure Source Data [file 44320_2025_98_MOESM12_ESM.zip › SD for EV and Appendix figures/Figure EV4/EV4E/shHNF4A-HNF4A.tif]

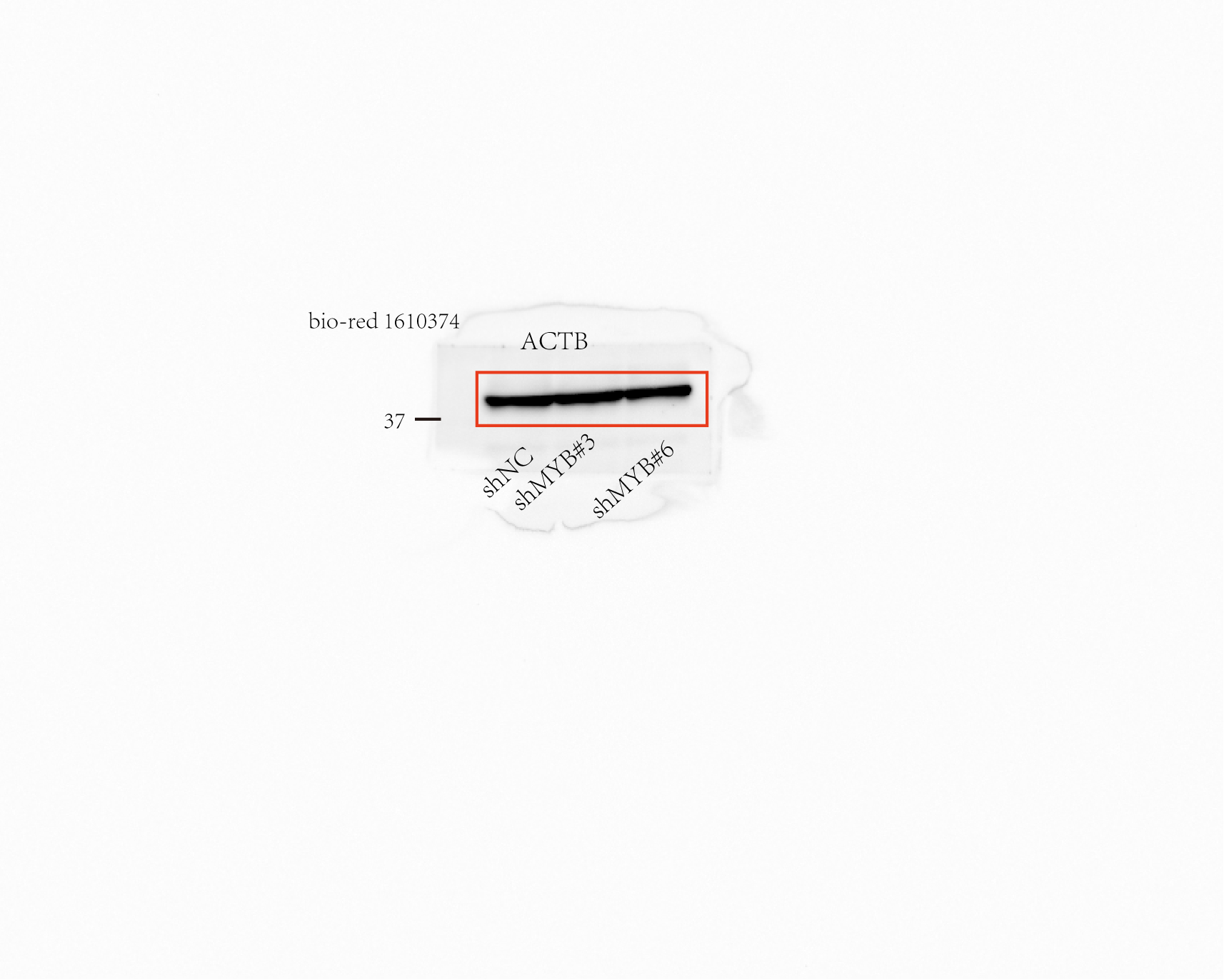

Supplement: Supplementary file 12 — EV and Appendix Figure Source Data [file 44320_2025_98_MOESM12_ESM.zip › SD for EV and Appendix figures/Figure EV5/EV5B/HT55 MYB-KD ACTB.tif]

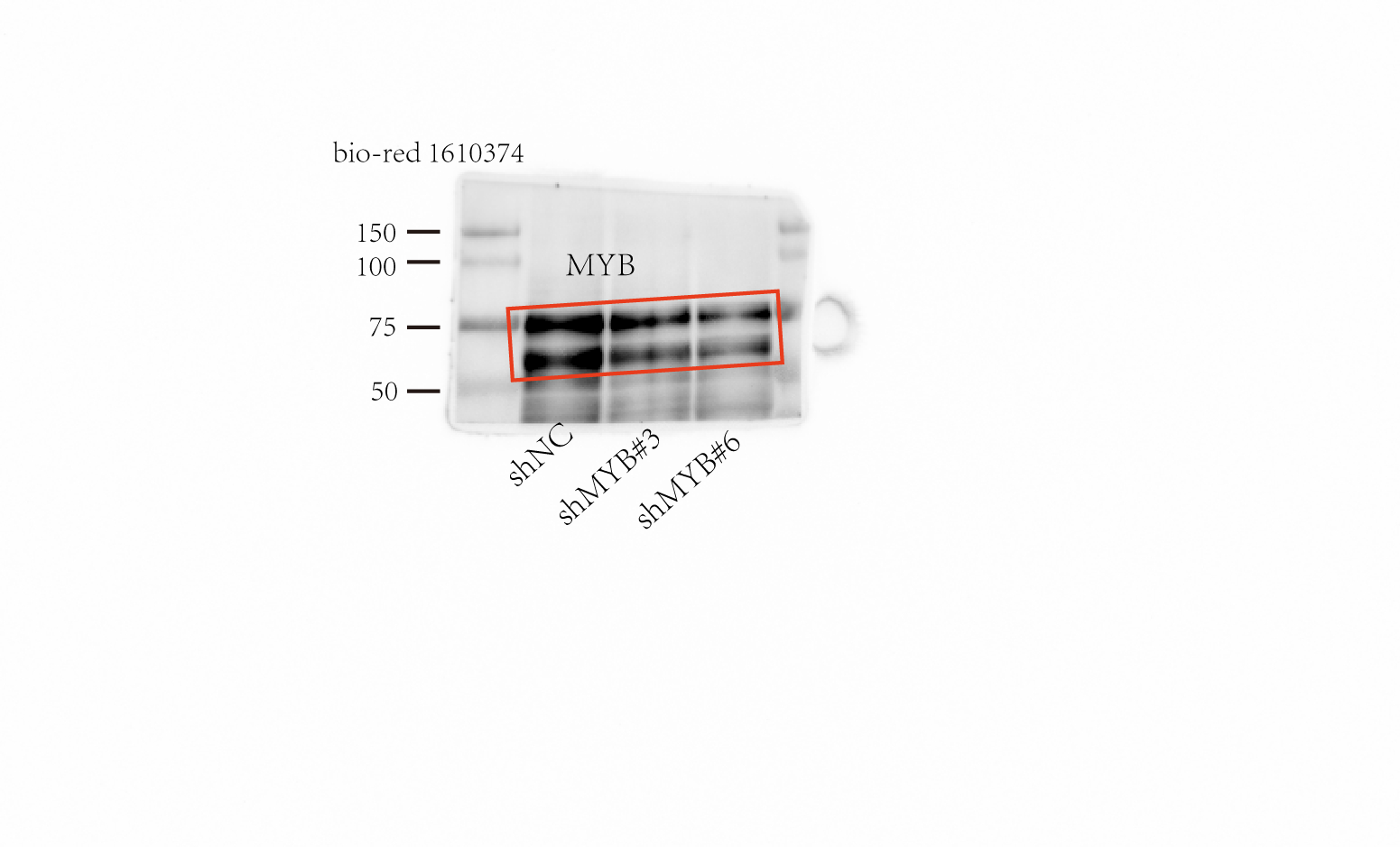

Supplement: Supplementary file 12 — EV and Appendix Figure Source Data [file 44320_2025_98_MOESM12_ESM.zip › SD for EV and Appendix figures/Figure EV5/EV5B/HT55 MYB-KD MYB.tif]

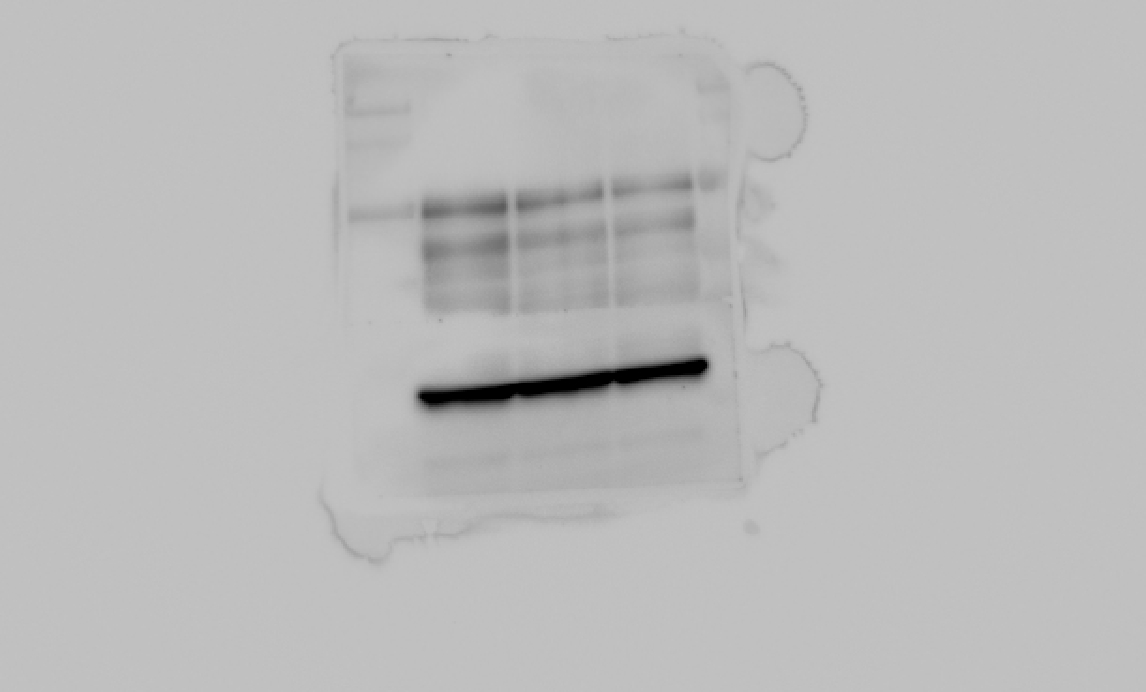

Supplement: Supplementary file 12 — EV and Appendix Figure Source Data [file 44320_2025_98_MOESM12_ESM.zip › SD for EV and Appendix figures/Figure EV5/EV5B/HT55.jpg]

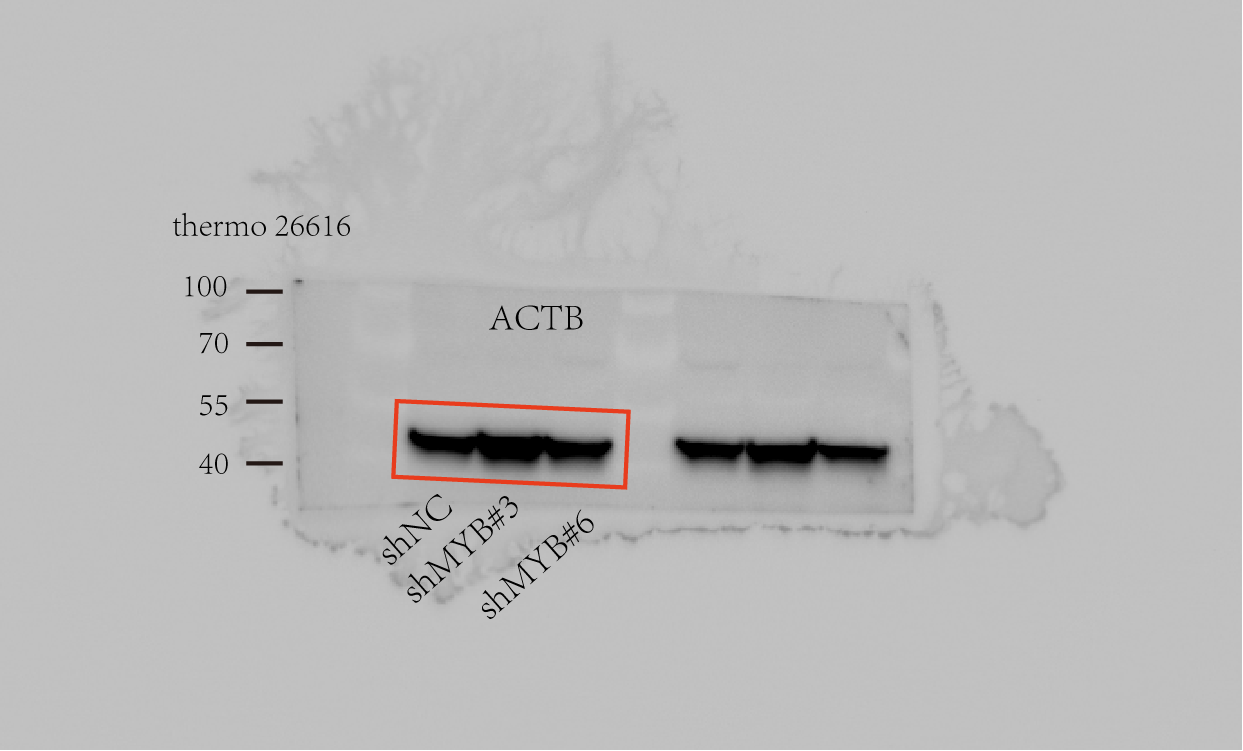

Supplement: Supplementary file 12 — EV and Appendix Figure Source Data [file 44320_2025_98_MOESM12_ESM.zip › SD for EV and Appendix figures/Figure EV5/EV5B/SNU719 MYB-KD ACTB.tif]

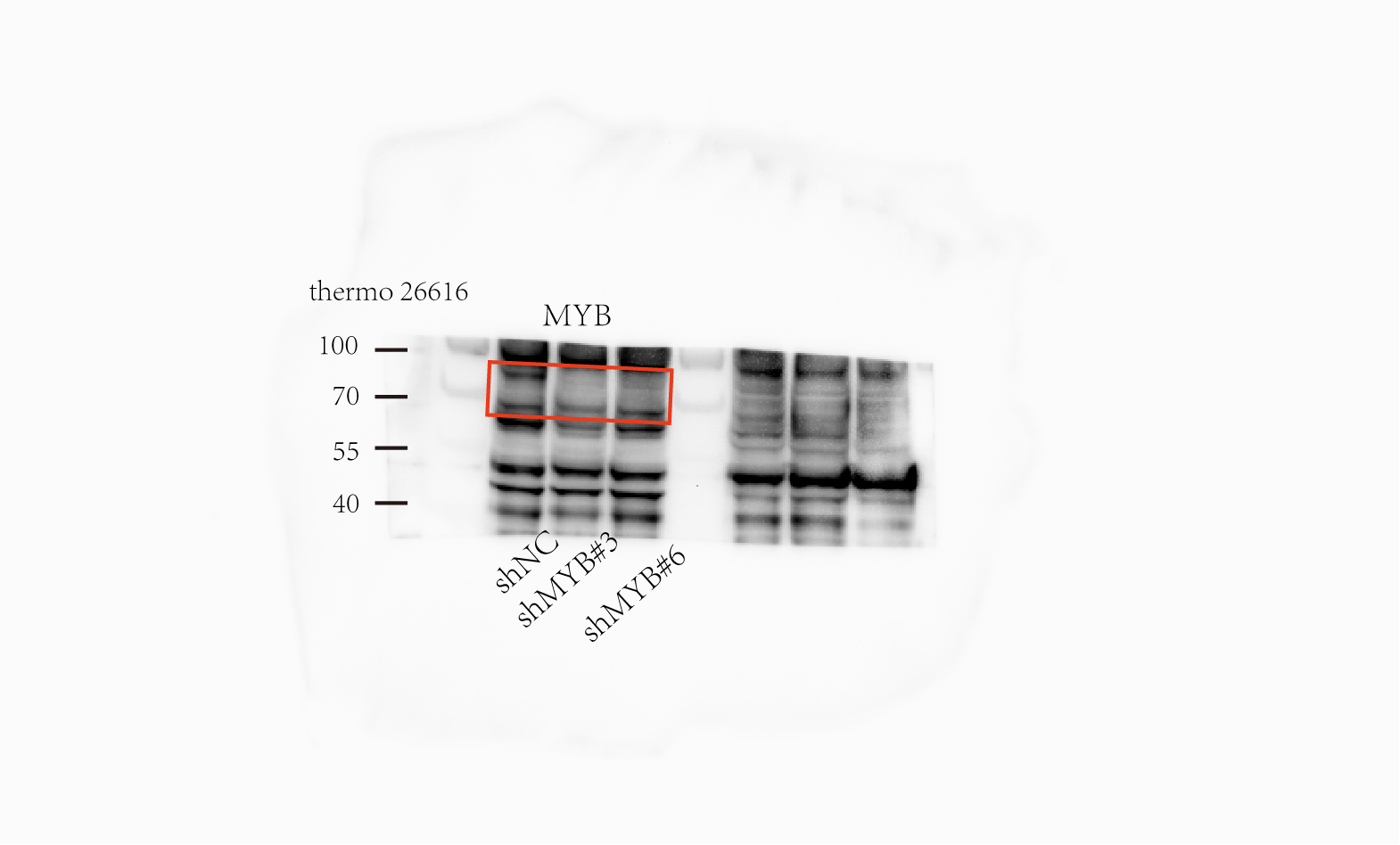

Supplement: Supplementary file 12 — EV and Appendix Figure Source Data [file 44320_2025_98_MOESM12_ESM.zip › SD for EV and Appendix figures/Figure EV5/EV5B/SNU719 MYB-KD MYB.tif]

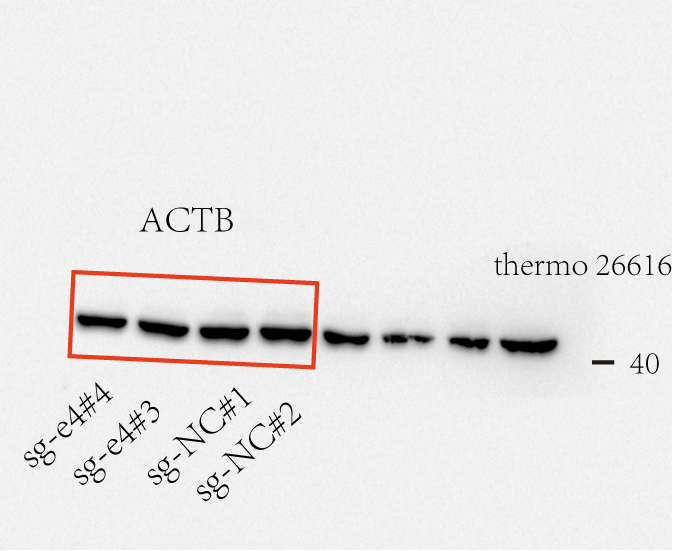

Supplement: Supplementary file 12 — EV and Appendix Figure Source Data [file 44320_2025_98_MOESM12_ESM.zip › SD for EV and Appendix figures/Figure EV5/EV5C/20221214_171012_0.3.02.tif]

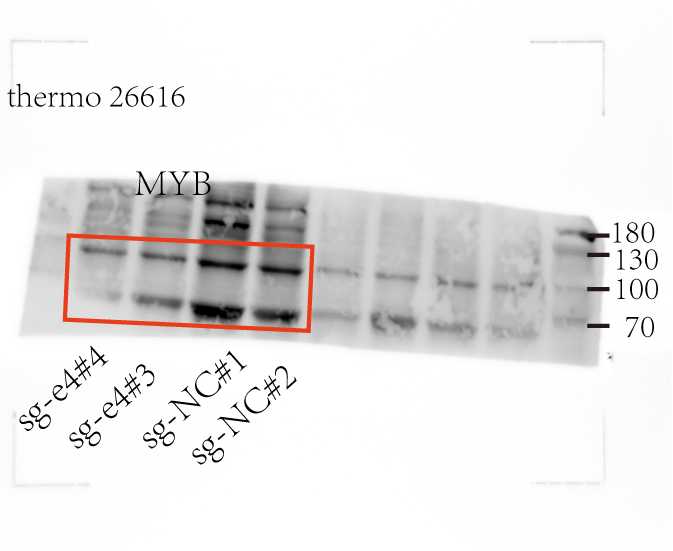

Supplement: Supplementary file 12 — EV and Appendix Figure Source Data [file 44320_2025_98_MOESM12_ESM.zip › SD for EV and Appendix figures/Figure EV5/EV5C/HT55-MYB.tif]

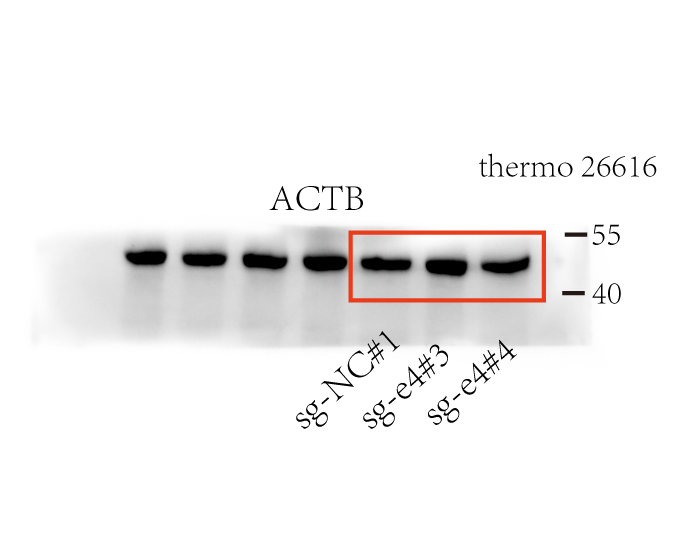

Supplement: Supplementary file 12 — EV and Appendix Figure Source Data [file 44320_2025_98_MOESM12_ESM.zip › SD for EV and Appendix figures/Figure EV5/EV5C/SNU719-ACTB.tif]

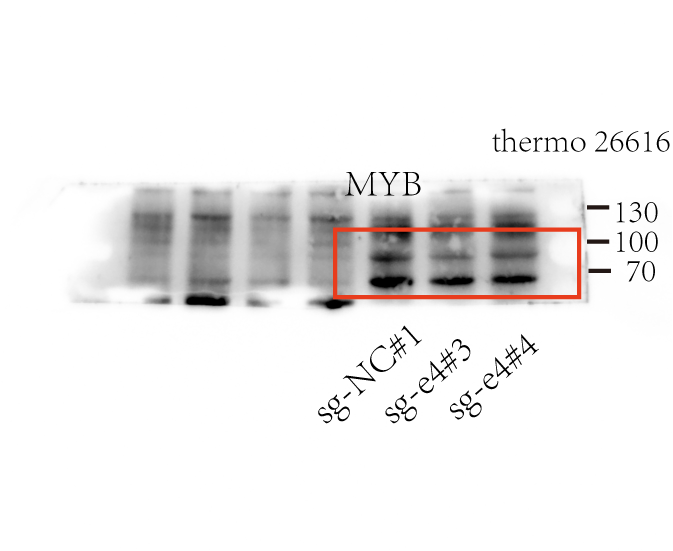

Supplement: Supplementary file 12 — EV and Appendix Figure Source Data [file 44320_2025_98_MOESM12_ESM.zip › SD for EV and Appendix figures/Figure EV5/EV5C/SNU719-MYB.tif]

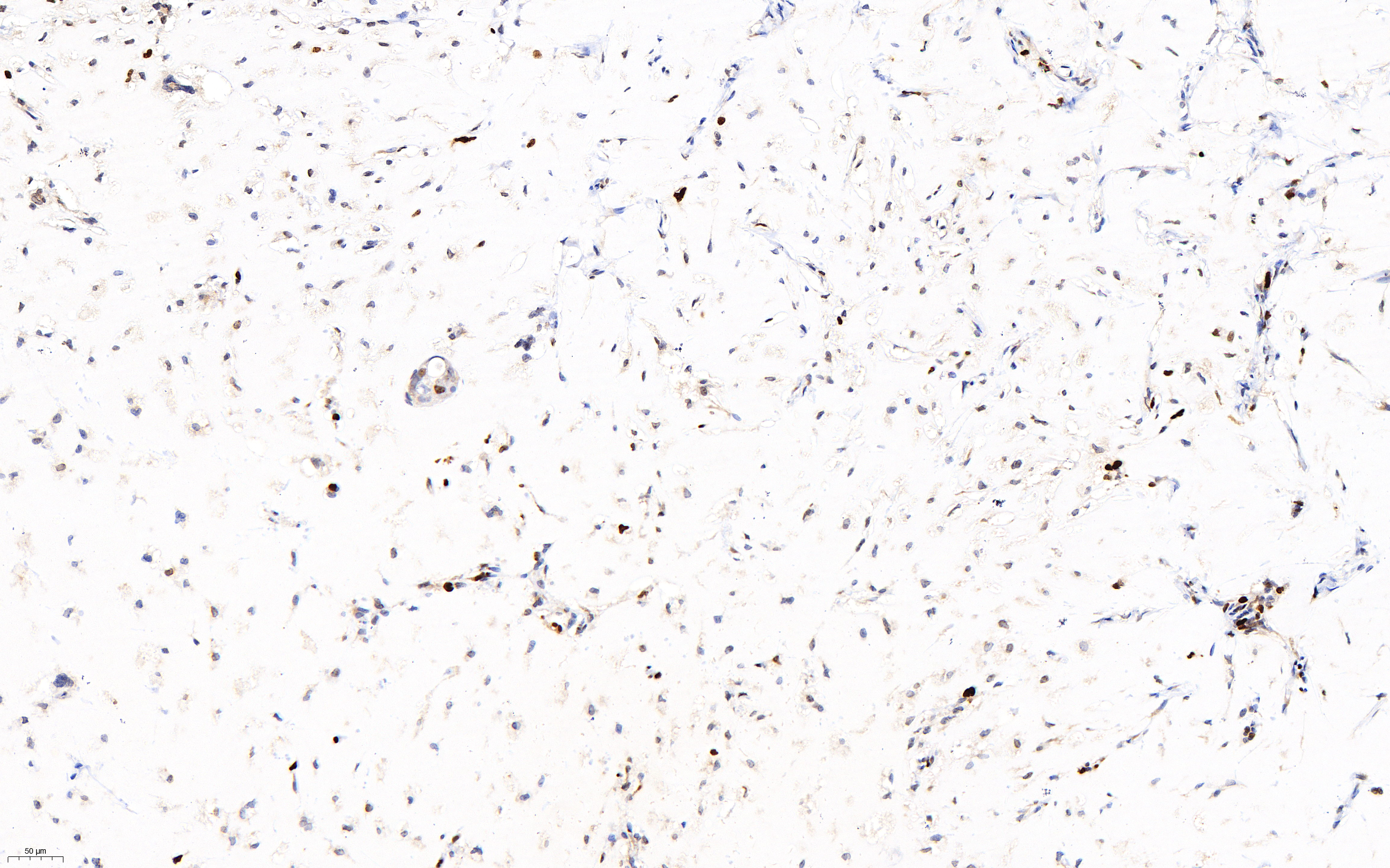

Supplement: Supplementary file 12 — EV and Appendix Figure Source Data [file 44320_2025_98_MOESM12_ESM.zip › SD for EV and Appendix figures/Figure EV5/EV5H/shMYB#3.tif]

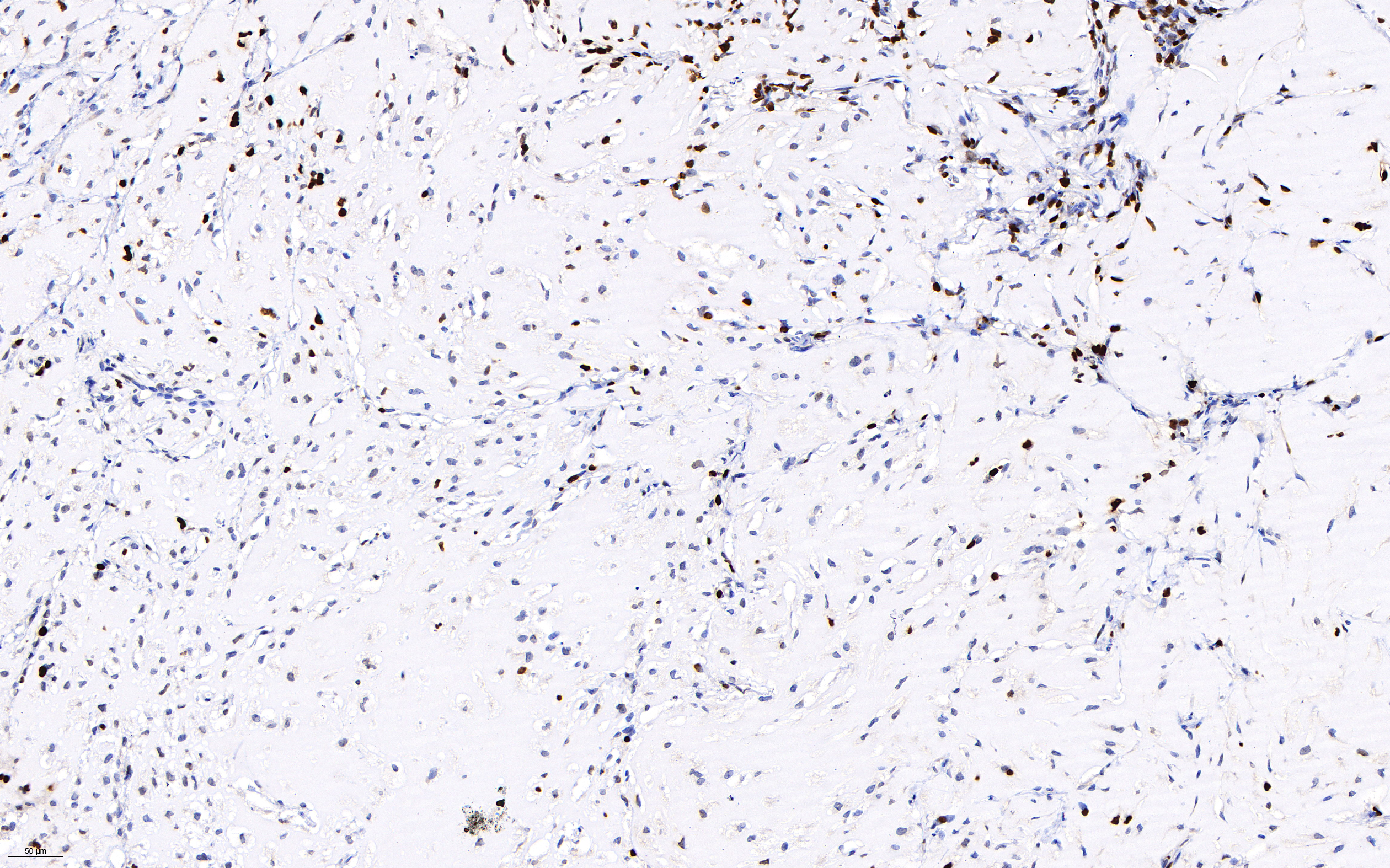

Supplement: Supplementary file 12 — EV and Appendix Figure Source Data [file 44320_2025_98_MOESM12_ESM.zip › SD for EV and Appendix figures/Figure EV5/EV5H/shMYB#6.tif]

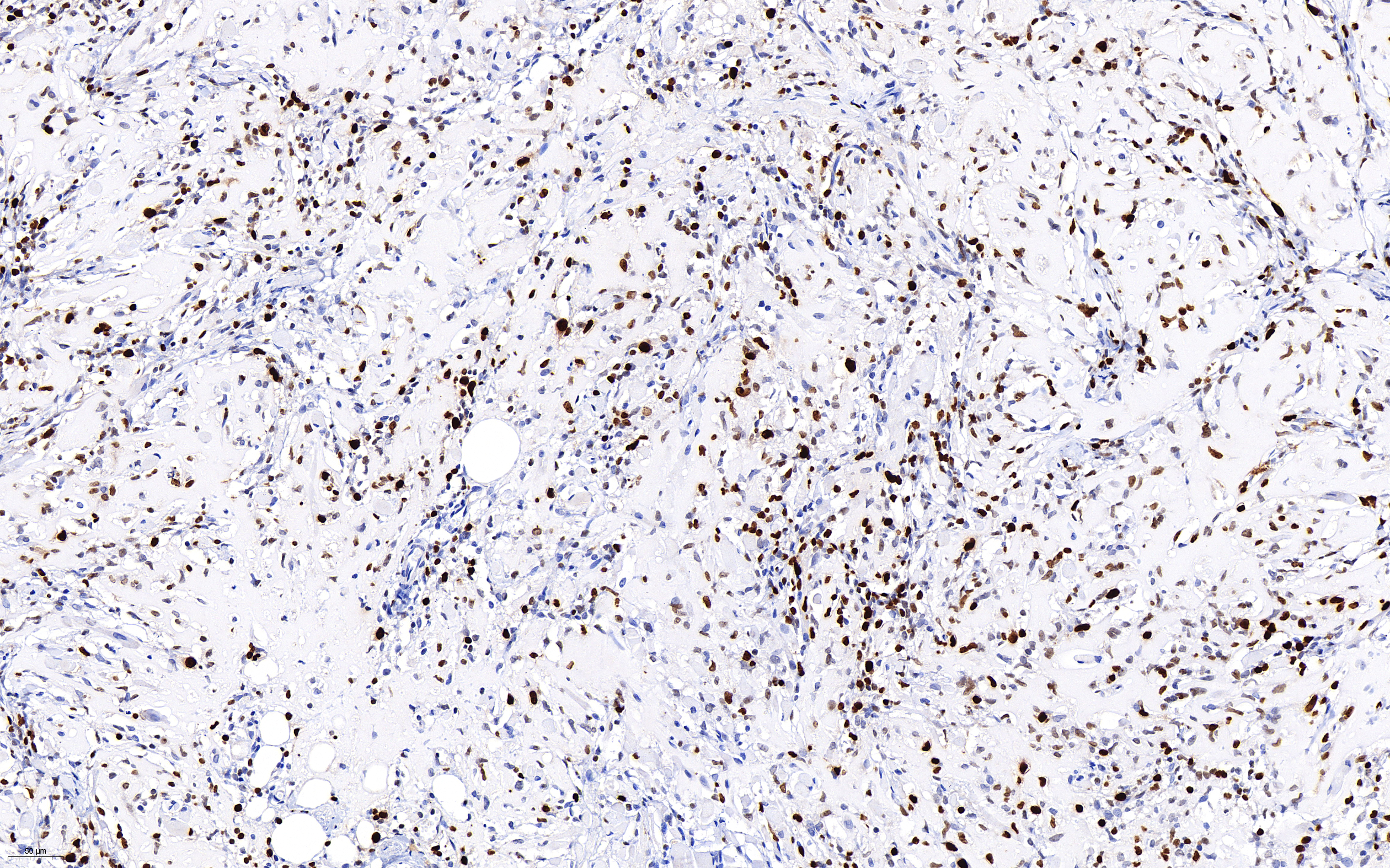

Supplement: Supplementary file 12 — EV and Appendix Figure Source Data [file 44320_2025_98_MOESM12_ESM.zip › SD for EV and Appendix figures/Figure EV5/EV5H/shNC.tif]
