# Supplementary material for: Systemic genome-epigenome analysis captures a lineage-specific super-enhancer for MYB in gastrointestinal adenocarcinoma
Source: Mol Syst Biol. 2025 Apr 15;21(6):696–719. doi: 10.1038/s44320-025-00098-1 (PMC12130324; doi:10.1038/s44320-025-00098-1)
Supplement: Supplementary file 13 — Expanded View Figures [file 44320_2025_98_MOESM13_ESM.pdf]

Expanded View Figures

Figure EV1. The gastrointestinal specificity of *MYB*-SE.

(**A**) Venn diagram showing the overlap between oncogenes associated with SEs and upregulated genes with physical interactions with SEs in gastrointestinal adenocarcinoma. (**B**) Venn diagram showing the shared SEs and their associated genes between gastrointestinal cancer cell lines and tumor samples, with oncogenes highlighted in red. (**C**) ChIP-seq signal tracks showing the enrichment of H3K27ac at the *MYB*-SE locus. The blue denoted COREAD tracks and red denoted STAD tracks; The cell line information provides a detailed description of the cell lines depicted in Fig. 1F. (**D**) Focal duplications harboring *MYB*-SE, shorter than 1 Mb in length, were selected for further analysis across 37 cancer types in the PCAWG project. (**E**) H3K27ac ChIP-seq tracks at the *MYB* locus in cell lines representing each cancer type. ESAD: esophageal adenocarcinoma; BLCA: bladder cancer; PADD: pancreatic adenocarcinoma; LIHC: liver hepatocellular carcinoma; UCEC: uterine corpus endometrial carcinoma; LUAD: lung adenocarcinoma; BRCA: breast cancer; PRAD: prostate adenocarcinoma; LAML: acute myeloid leukemia. (**F**) ChIP-seq signal tracks of H3K27ac at the *Myb* and *Myc* loci in mouse CRC cell lines. The region of *MYB*-SE is conserved between humans and mice except the enhancer e6. However, the activity of human gastrointestinal adenocarcinoma cancer-specific super-enhancer of *MYB* (converted to mm10 genome) is repressed in this region in mouse CRC cell lines. (**G**) Expression levels of *Myb* and *Myc* in mouse CRC cell lines, based on RNA-seq data. (**H**) The expression of *MYB* in cancer tissues and normal tissues, from the TCGA and GTEx databases, was analyzed using Gene Expression Profiling Interactive Analysis 2 (GEPIA2) (Tang et al, 2019). The exact *P*-value were not provided in GEPIA2. The *P*-value was determined by two-sided Student's *t*-test. ***$P \leq 0.001$; Box plots represent the distribution of MYB expression across different groups. The center line within each box denotes the median. The lower and upper bounds of the box indicate the first quartile (Q1, 25th percentile) and third quartile (Q3, 75th percentile), respectively. The whiskers extend to the minimum and maximum values within 1.5× interquartile range (IQR) from Q1 and Q3. Individual points represent paired data and are connected by lines, while potential outliers beyond 1.5× IQR are displayed as separate dots. (**I**) The expression of *MYB* in COREAD or STAD samples and their corresponding pair-matched normal tissues. The value for the y-axis is $\log_2$ transformed (count+1). $N = 32$ for COREAD or STAD. NS: no significance. Box plots represent the distribution of *MYB* expression across different groups. The center line within each box denotes the median. The lower and upper bounds of the box indicate the first quartile (Q1, 25th percentile) and third quartile (Q3, 75th percentile), respectively. The whiskers extend to the minimum and maximum values within 1.5× interquartile range (IQR) from Q1 and Q3. Individual points represent paired data and are connected by lines, while potential outliers beyond 1.5× IQR are displayed as separate dots. (**J**) Correlation between *MYB* expression and *MYB* copy number, calculated using Spearman's rank correlation coefficient based on the data from CCLE.

© The Author(s)

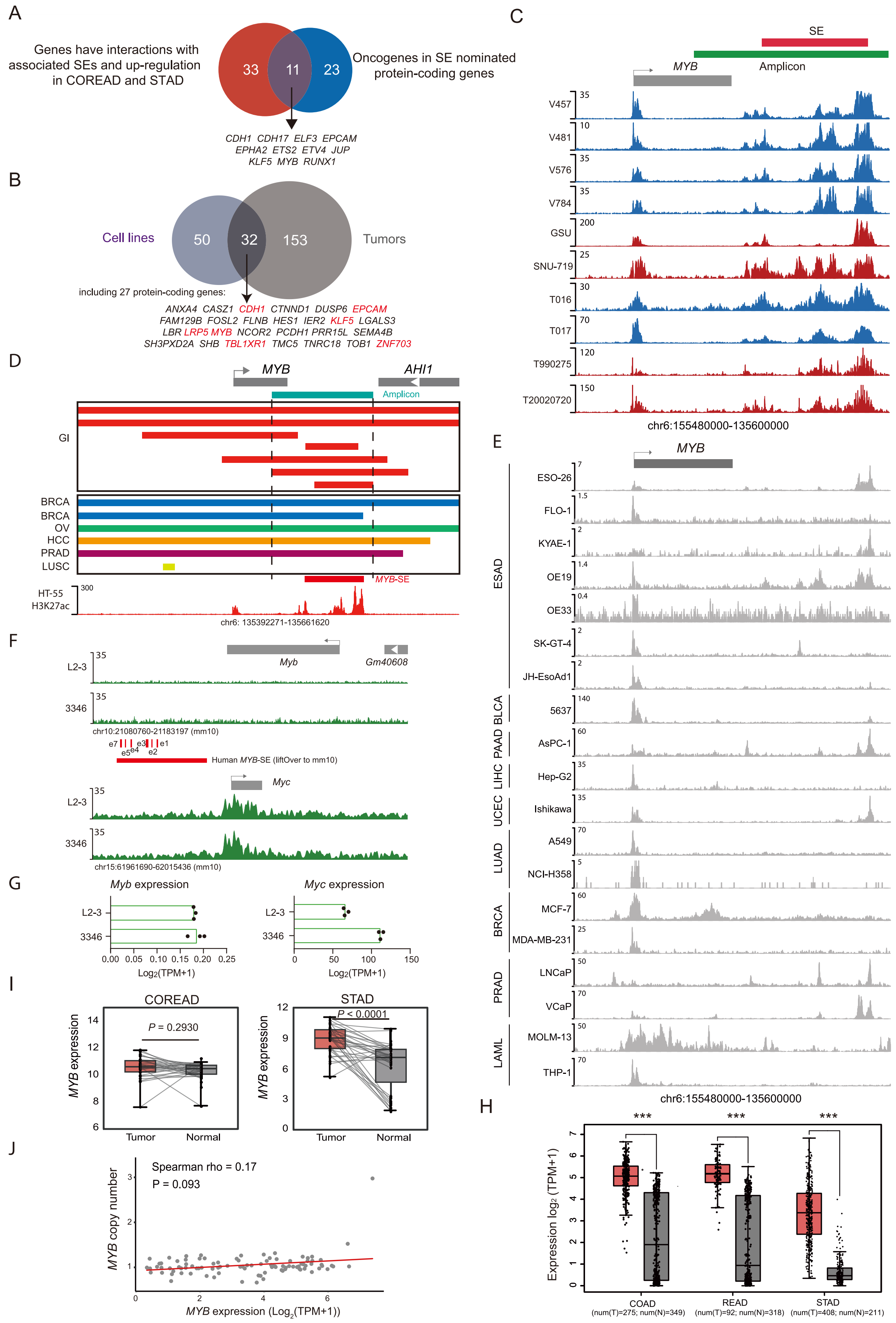
A
Genes have interactions with associated SEs and up-regulation in COREAD and STAD
33
11
23
Oncogenes in SE nominated protein-coding genes
CDH1 CDH17 ELF3 EPCAM
EPHA2 ETS2 ETV4 JUP
KLF5 MYB RUNX1
B
Cell lines
50
32
153
Tumors
including 27 protein-coding genes:
ANXA4 CASZ1 CDH1 CTNND1 DUSP6 EPCAM
FAM129B FOSL2 FLNB HES1 IER2 KLF5 LGALS3
LBR LRP5 MYB NCOR2 PCDH1 PRR15L SEMA4B
SH3PXD2A SHB TBL1XR1 TMC5 TNRC18 TOB1 ZNF703
C
SE
MYB
Amplicon
V457
V481
V576
V784
GSU
SNU-719
T016
T017
T990275
T20020720
chr6:155480000-135600000
D
MYB
AHI1
Amplicon
GI
BRCA
BRCA
OV
HCC
PRAD
LUSC
MYB-SE
HT-55 H3K27ac
chr6: 135392271-135661620
E
MYB
ESAD
ESO-26
FLO-1
KYAE-1
OE19
OE33
SK-GT-4
JH-EsoAd1
BLCA
5637
PAAD
AsPC-1
LIHC
Hep-G2
UCEC
Ishikawa
LUAD
A549
NCI-H358
BRCA
MCF-7
MDA-MB-231
PRAD
LNCaP
VCaP
LAML
MOLM-13
THP-1
chr6:155480000-135600000
F
L2-3
3346
Myb
Gm40608
chr10:21080760-21183197 (mm10)
e7 e5 e4 e3 e2 e1
Human MYB-SE (liftOver to mm10)
Myc
chr15:61961690-62015436 (mm10)
G
Myb expression
Myc expression
Log2(TPM+1)
H
Expression log2 (TPM+1)
***
COAD
(num(T)=275; num(N)=349)
READ
(num(T)=92; num(N)=318)
STAD
(num(T)=408; num(N)=211)
I
COREAD
P = 0.2930
STAD
P < 0.0001
MYB expression
Tumor
Normal
J
Spearman rho = 0.17
P = 0.093
MYB copy number
MYB expression (Log2(TPM+1))

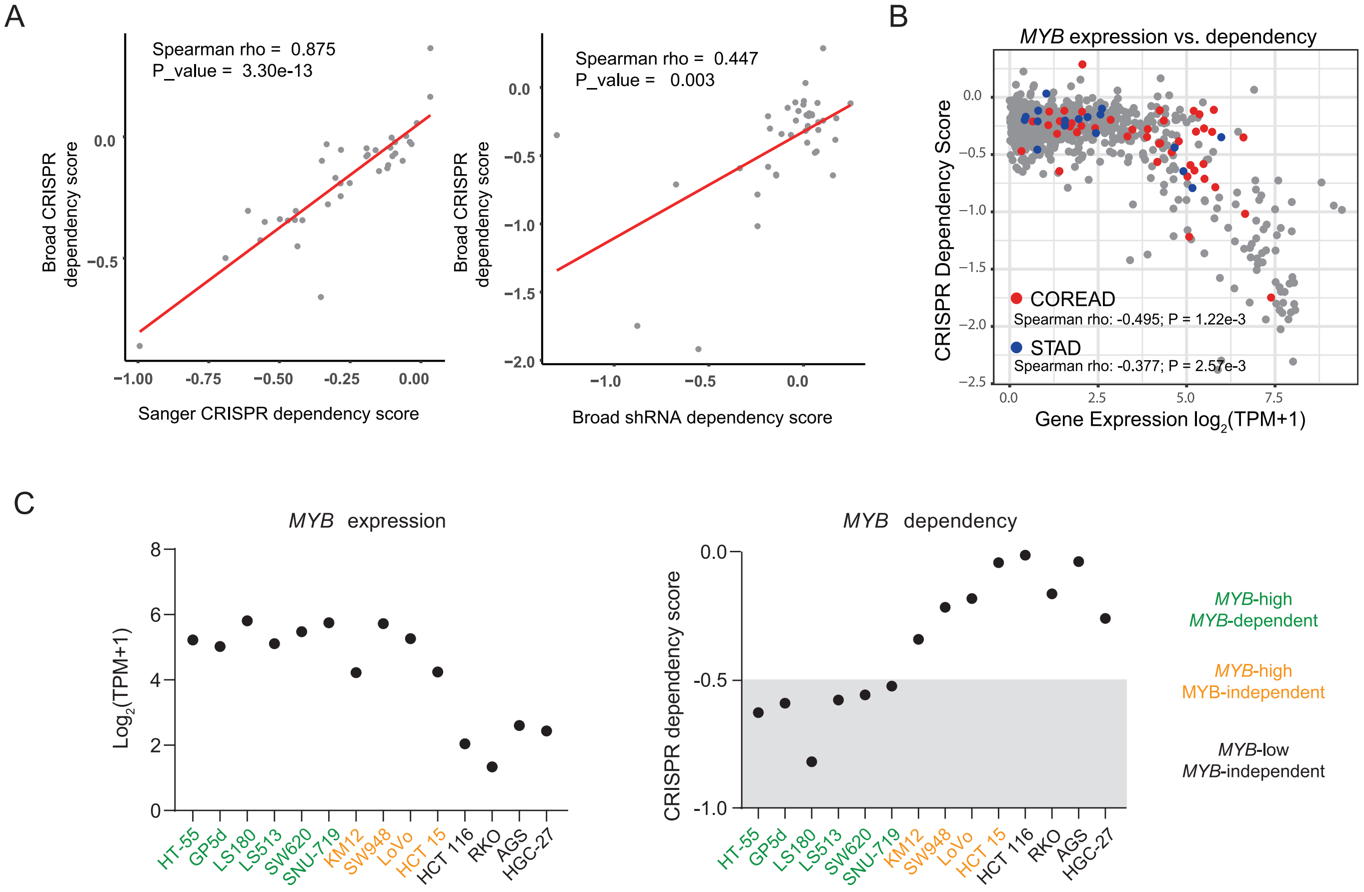

Figure EV2. Functional significance of *MYB* in gastrointestinal adenocarcinoma.

(**A**) Correlation between *MYB* dependency scores from DepMap and results from the Sanger Institute CRISPR screen (left) or the Broad Institute shRNA screen (right), calculated using Spearman's rank correlation coefficient based on the data from CCLE. (**B**) Correlation between *MYB* expression and dependency scores across all cancer cell lines in DepMap, calculated using Spearman's rank correlation coefficient based on the data from CCLE. The results of COREAD and STAD cell lines were highlighted in red and blue, respectively. (**C**) The expression levels and dependency scores of *MYB* in the indicated cell lines based on DepMap. Source data are available online for this figure.

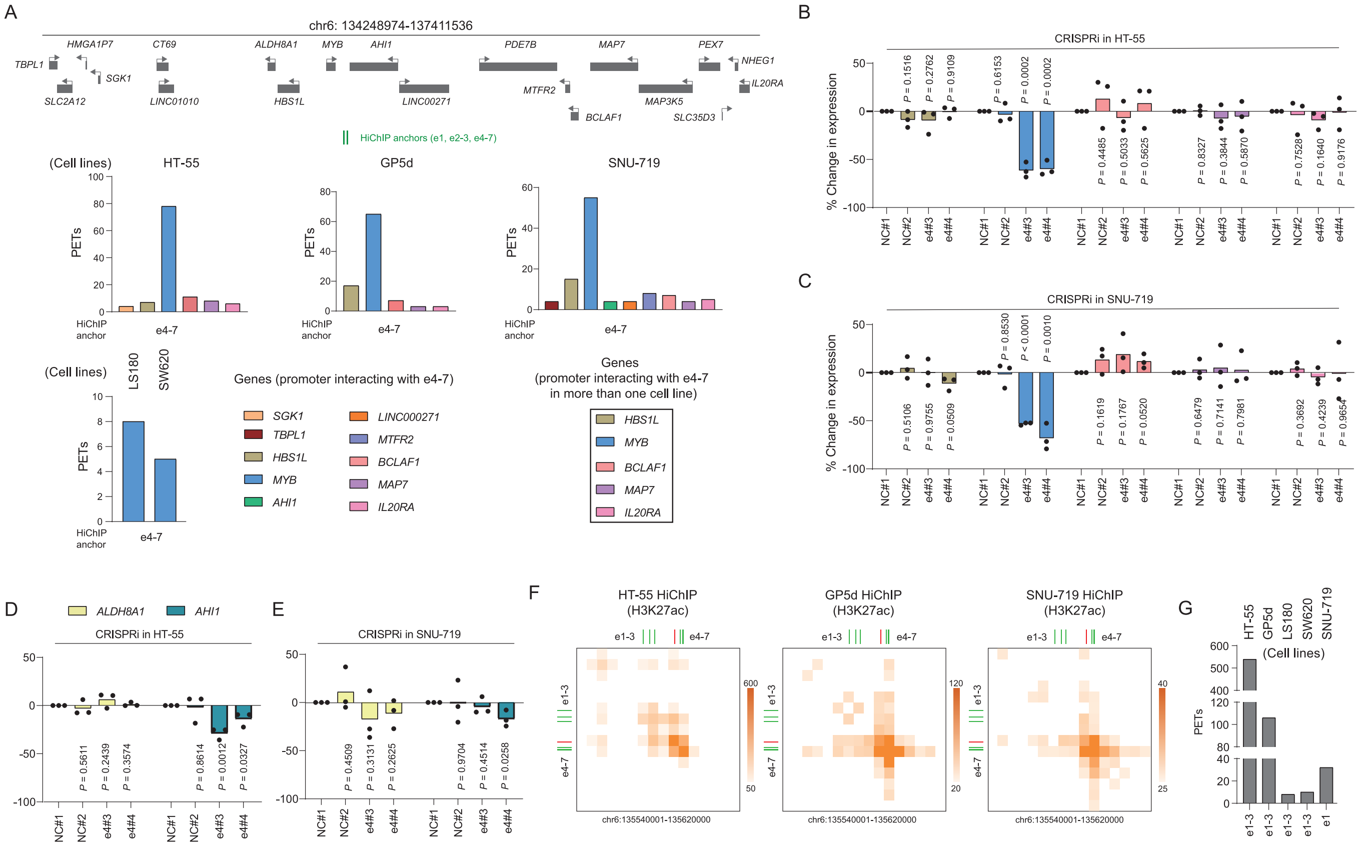

Figure EV3. The enhancer e4 is the predominant element of the *MYB*-SE.

(**A**) The number of paired-end tags (PETs) that connecting e4–7 anchor to the promoters of candidate genes in five cell lines, based on H3K27ac HiChIP data. (**B**, **C**) The expression of candidate genes following CRISPRi targeting e4 in HT-55 (**B**) and SNU-719 (**C**) cells. The genes were annotated with the same color as (**A**). All values are mean ± S.D. The *P*-value was determined by two-sided Student's *t*-test. (**D**, **E**) The expression of genes adjacent to *MYB* following CRISPRi targeting e4 in HT-55 (**D**) and SNU-719 (**E**) cells. The genes were annotated with the same color as (**A**). All values are mean ± S.D. The *P*-value was determined by two-sided Student's *t*-test. (**F**) The contact heatmap based on H3K27ac HiChIP data revealed that e4 interacted with other candidate enhancers within the *MYB*-SE in HT-55, GP5d, and SNU-719 cells. (**G**) The number of PETs that connecting e4–7 anchor to e1–3 or e1 anchor based on H3K27ac HiChIP data in five cell lines. Source data are available online for this figure.

© The Author(s)

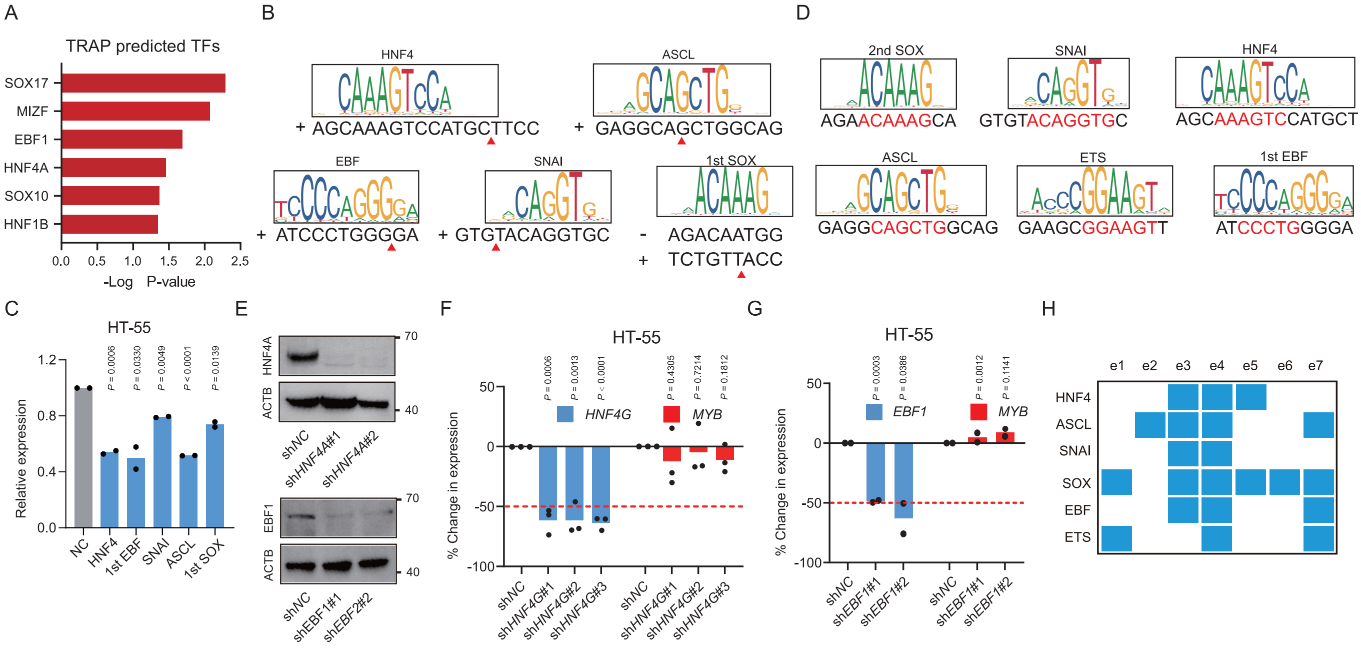

Figure EV4. Candidate transcription factors regulating *MYB* expression through interactions with e4.

(**A**) Transcription factors predicted to bind to e4 based on TRAP motif analysis; the *P*-value was determined by Benjamini-Hochberg multiple comparison correction method. (**B**) Demonstration of the motifs to be disrupted by CRISPR-Cas9. Red triangles indicated the cutting sites. (**C**) The expression of *MYB* in HT-55 cells following CRISPR-Cas9 based disruption of the identified motifs. $N = 2$. All values are mean ± S.D. The *P*-value was determined by two-sided Student's *t*-test. (**D**) Demonstration of the motifs to be deletion by site-specific mutagenesis for the Luciferase assay. Red sequences indicated the targets of deletion. (**E**) Western blotting showing a decrease in HNF4A and EBF1 protein levels upon *HNF4A* or *EBF1* knockdown, respectively. (**F**) The expression of *HNF4G* and *MYB* upon *HNF4G* knockdown. NC, non-targeted control. $N = 3$. All values are mean ± S.D. The *P*-value was determined by two-sided Student's *t*-test. (**G**) The expression of *EBF1* and *MYB* upon *EBF1* knockdown. NC, non-targeted control. $N = 3$. All values are mean ± S.D. The *P*-value was determined by two-sided Student's *t*-test. (**H**) Presence of the candidate functional motifs in the e1–e7 enhancers. Source data are available online for this figure.

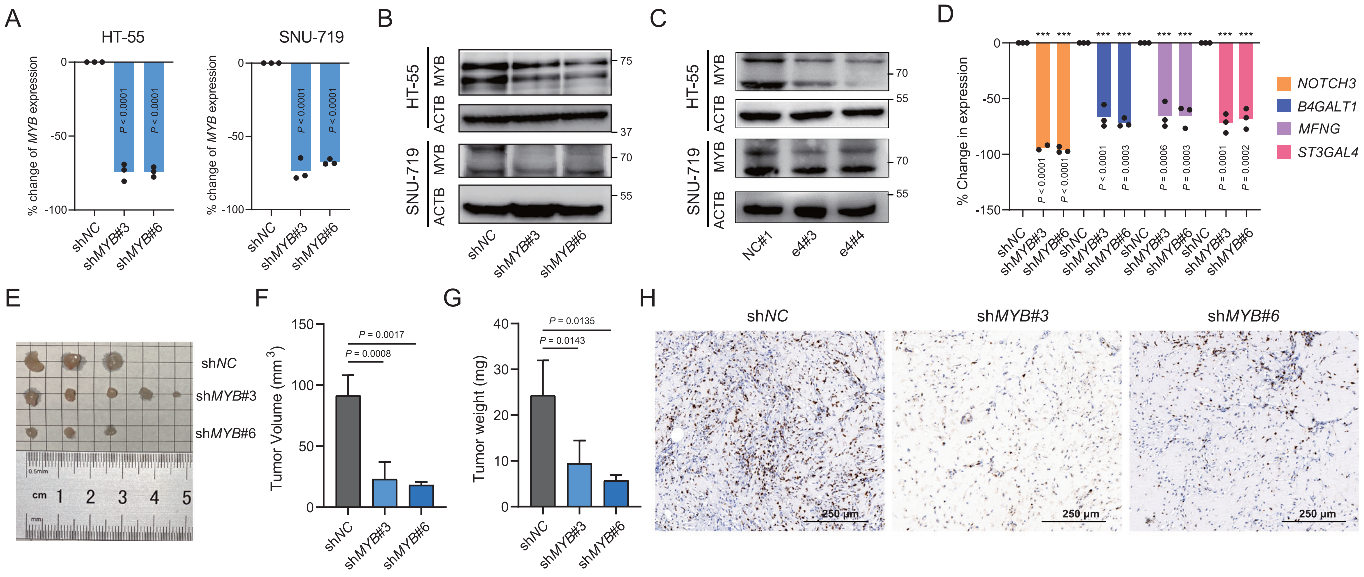

Figure EV5. Repression of *MYB* expression in vivo inhibited the development of gastrointestinal adenocarcinoma.

(**A**) The expression of *MYB* upon *MYB* knockdown in HT-55 and SNU-719 cells. $N = 3$. All values are mean ± S.D. The *P*-value was determined by two-sided Student's *t*-test. (**B**, **C**) Western blotting showing the decreased MYB protein upon *MYB* knockdown (**B**) or CRSPRi targeting e4 (**C**) in HT-55 and SNU-719 cells. The ACTB protein served as a loading control. (**D**) RT-qPCR analysis demonstrated the alterations in Notch signaling-related genes following *MYB* knockdown. $N = 3$. All values are mean ± S.D. The *P*-value was determined by two-sided Student's *t*-test. (**E–G**) The growth of the xenografts derived from HT-55 cells was significantly inhibited following *MYB* knockdown. $N = 3–5$. All values are mean ± S.D. The *P*-value was determined by two-sided Student's *t*-test. (**H**) Immunohistochemical staining for Ki-67 in xenograft samples following *MYB* knockdown in HT-55 cells. Scale bar = 250 µm. Source data are available online for this figure.

© The Author(s)
